# Engineering cuproptosis with nanomedicine: Design, combination therapy, and translation in cancer

**DOI:** 10.1016/j.mtbio.2026.103466

**Published:** 2026-07-15

**Authors:** Dongsheng Fan, Jianxin Liu, Fang Ren, Long Wang, Jing Wei, Dalian Qin, Xiaogang Zhou, Jianming Wu, Xiao Zou, Anguo Wu

**Affiliations:** a Department of Pharmacy, The First Affiliated Hospital of Guizhou University of Traditional Chinese Medicine, Guiyang, 550000, China; b School of Pharmaceutical Sciences, Sino-Pakistan Center on Traditional Chinese Medicine, China-Pakistan International Science and Technology Innovation Cooperation Base for Ethnic Medicine Development in Hunan Province, Hunan University of Medicine, Huaihua, 418000, China; c Chongqing Key Laboratory of Sichuan-Chongqing Co-construction for Diagnosis and Treatment of Infectious Diseases Integrated Traditional Chinese and Western Medicine, Department of Laboratory Medicine, Chongqing Hospital of Traditional Chinese Medicine, Chongqing, 400016, China; d Sichuan Key Medical Laboratory of New Drug Discovery and Druggability Evaluation, Key Laboratory of Medical Electrophysiology of Ministry of Education, The Affiliated Hospital of Southwest Medical University, School of Pharmacy, Southwest Medical University, Luzhou, 646000, China; e Department of Ophthalmology, The Affiliated Hospital of Southwest Medical University, Luzhou, 646000, China; f Department of Pharmacy, Chengdu Wenjiang District People's Hospital, Chengdu, 611130, China; g Institute of Traditional Chinese Medicine Health Industry, China Academy of Chinese Medical Sciences, Nanchang, 330038, China

**Keywords:** Cuproptosis, Nanomedicine, Copper homeostasis, Combination therapy, Cancer

## Abstract

Cuproptosis is a distinct form of regulated cell death triggered by copper and characterized by the aggregation of lipoylated mitochondrial proteins, destabilization of iron–sulfur cluster proteins, and proteotoxic stress. Unlike nonspecific copper toxicity, cuproptosis depends on a defined biochemical context shaped by FDX1-associated protein lipoylation, oxidative carbon metabolism, and mitochondrial substrate competence. These features may create a therapeutic vulnerability in tumors with high oxidative phosphorylation, but they also impose substantial translational challenges. Free copper ions and conventional copper ionophores often lack the tumor selectivity, pharmacological control, and safety required for systemic use, and copper exposure alone is insufficient unless it can be converted into sustained mitochondrial stress in susceptible tumor cells. Nanomedicine can provide spatiotemporal and chemical control over tumor copper stress. By enabling tumor-selective accumulation, stimulus-responsive activation, mitochondrial targeting, and modulation of copper homeostasis, nanoplatforms may help convert cuproptosis from a biochemical phenomenon into a therapeutically tractable strategy. These capabilities create opportunities to integrate cuproptosis with broader tumor vulnerabilities, including redox imbalance, metabolic plasticity, and altered immune states. This Review examines the molecular basis of cuproptosis and tumor susceptibility, the design and combination strategies of cuproptosis-oriented nanomedicines, and the translational challenges that must be addressed to move the field beyond proof of concept. Future advances are likely to depend on precise control of copper bioavailability and mitochondrial targeting, together with biomarker-guided patient selection and clinically tractable platform design.

## Highlights

• Cuproptosis exploits FDX1–lipoylation-dependent mitochondrial vulnerability.
• Nanomedicine controls copper bioavailability, release, and mitochondrial access.
• Copper-supplying and copper-depleting strategies are mechanistically distinct.
• Rational combinations target redox, metabolic, and immune vulnerabilities.

## Graphical abstract

Cuproptosis is a copper-dependent form of regulated cell death driven by mitochondrial copper stress, FDX1-associated copper activation and protein lipoylation, aggregation of lipoylated mitochondrial proteins, Fe–S protein destabilization, and proteotoxic injury. Tumor susceptibility is shaped by copper homeostasis and metabolic state, while nanomedicine can enhance pathway control through copper delivery and copper-ionophore-loaded nanoparticles, stimulus-responsive release, mitochondrial targeting, GSH depletion, copper-exporter modulation, modulation of the FDX1–lipoylation axis, metabolic reprogramming toward oxidative phosphorylation, and activation of immunogenic signaling.

**Figure:**
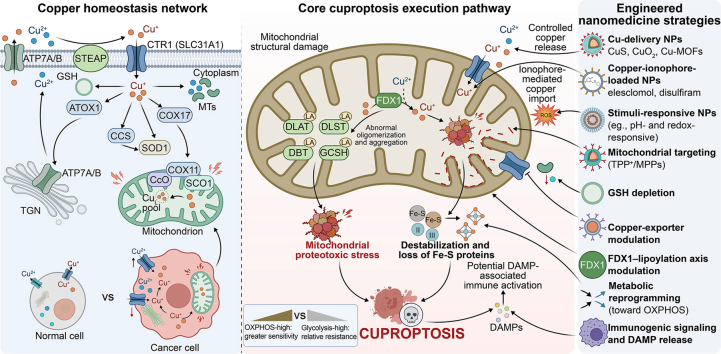

## 1 Introduction

Copper (Cu) is an essential micronutrient with important roles in cellular metabolism, but its redox activity also makes it potentially toxic when homeostasis is disrupted [1,2]. As a cofactor for enzymes involved in mitochondrial respiration, antioxidant defense, and connective tissue remodeling, copper contributes to core biological processes in both normal tissues and cancer [1,2]. To balance essential copper use against toxicity, mammalian cells regulate copper through transporters, chaperones and exporters, including copper transporter 1 (CTR1; encoded by SLC31A1), antioxidant 1 copper chaperone (ATOX1), copper chaperone for superoxide dismutase (CCS), cytochrome *c* oxidase copper chaperone 17 (COX17), and ATPase copper transporting alpha/beta (ATP7A/ATP7B) [[2], [3], [4]]. Disruption of copper homeostasis has been reported across multiple cancers, with elevated copper levels detected in the serum and tumor tissues of patients with breast, lung, colorectal, and liver cancers [5,6]. Copper also contributes actively to oncogenic signaling rather than serving only as a metabolic cofactor, with reported links to BRAF–MEK–ERK signaling, hypoxia-inducible factor 1-alpha (HIF-1α)-driven angiogenesis, and LOX-mediated extracellular matrix remodeling during metastasis [5,[7], [8], [9]].

In 2022, Tsvetkov et al. defined cuproptosis as a form of copper-dependent cell death driven by copper binding to lipoylated tricarboxylic acid cycle proteins. This interaction promotes protein aggregation, loss of iron–sulfur cluster proteins, and lethal proteotoxic stress [10]. Ferredoxin 1 (FDX1) was identified as an important upstream regulator of this pathway. It is required for efficient protein lipoylation and has been proposed to facilitate copper reduction [10,11]. Cuproptosis is genetically and pharmacologically distinct from apoptosis, ferroptosis, necroptosis, and pyroptosis [10,[12], [13], [14], [15]]. Because cuproptosis depends on mitochondrial metabolism, tumors with high oxidative phosphorylation activity may be particularly sensitive, creating a possible metabolic therapeutic window, especially in therapy-resistant or stem-like states [10,16,17]. Emerging evidence further suggests that cuproptosis-inducing strategies may trigger hallmarks of immunogenic cell death, including calreticulin exposure and the release of high mobility group box 1 (HMGB1) and adenosine triphosphate (ATP), thereby potentially influencing antitumor immune responses [18,19].

Despite this promise, the therapeutic application of cuproptosis faces substantial pharmacological challenges. Copper is not a conventional cytotoxic agent for systemic use because its redox activity creates a narrow therapeutic window, and systemic overload can cause hepatotoxicity, nephrotoxicity, and neurotoxicity, as illustrated by Wilson disease [3,20]. Elesclomol and disulfiram (DSF), the two best-known copper ionophores, show that enforced intracellular copper accumulation can suppress tumor growth, but they also illustrate the limitations of this strategy. Elesclomol did not improve progression-free survival in an unselected phase III melanoma population, although subgroup analyses suggested that biological context may influence response. DSF is rapidly metabolized and chemically unstable, limiting the formation and persistence of active copper complexes at tumor sites [[21], [22], [23]]. More broadly, free copper ions and classical copper ionophores do not provide sufficient spatial, temporal, or chemical control over copper stress to target tumors effectively while limiting damage to normal tissues. Nanomedicine offers a potential route to address these limitations by improving tumor accumulation, reducing premature copper release, stabilizing copper or copper-ionophore species, and enabling controlled activation within tumor tissues [[24], [25], [26], [27], [28], [29], [30], [31]]. These capabilities are particularly relevant to cuproptosis because therapeutic efficacy depends not only on copper exposure, but also on mitochondrial copper bioavailability, copper redox state, and the ability to overcome intracellular buffering systems such as glutathione [10,24,25]. In this context, nanoplatforms may help convert cuproptosis from a biochemical phenomenon into a controllable therapeutic strategy, especially when copper delivery is integrated with metabolic modulation, redox disruption, immunotherapy, or other rational combination approaches [16,19,25,32].

Several recent reviews have summarized copper metabolism, the biology of cuproplasia and cuproptosis, cancer-associated mechanisms of cuproptosis, and copper-based nanomedicine [[28], [29], [30], [31],[33], [34], [35]]. These reviews have advanced the field by clarifying copper homeostasis, cuproptosis execution, copper-associated tumor biology, and representative nanotherapeutic strategies. However, most have focused primarily on mechanistic biology, disease associations, or material-based examples. In contrast, this Review adopts a mechanism-guided framework that connects biological insight, nanomedicine engineering, and translational development. Rather than simply cataloging copper-containing nanomaterials, we focus on how nanoplatforms can coordinate copper bioavailability, mitochondrial access, tumor metabolic state, redox buffering, FDX1-associated vulnerability, and antitumor immune activation to achieve controllable cuproptosis. We then examine the molecular determinants of cuproptosis and tumor susceptibility, the design principles and combination strategies of cuproptosis-oriented nanomedicines, and the translational barriers that must be overcome beyond proof of concept. By integrating mechanism, engineering, and translation, we aim to provide a coherent roadmap for developing biomarker-guided, manufacturable, and clinically translatable cuproptosis-oriented nanomedicines.

## 2 Molecular basis of cuproptosis

**Fig. 1:**
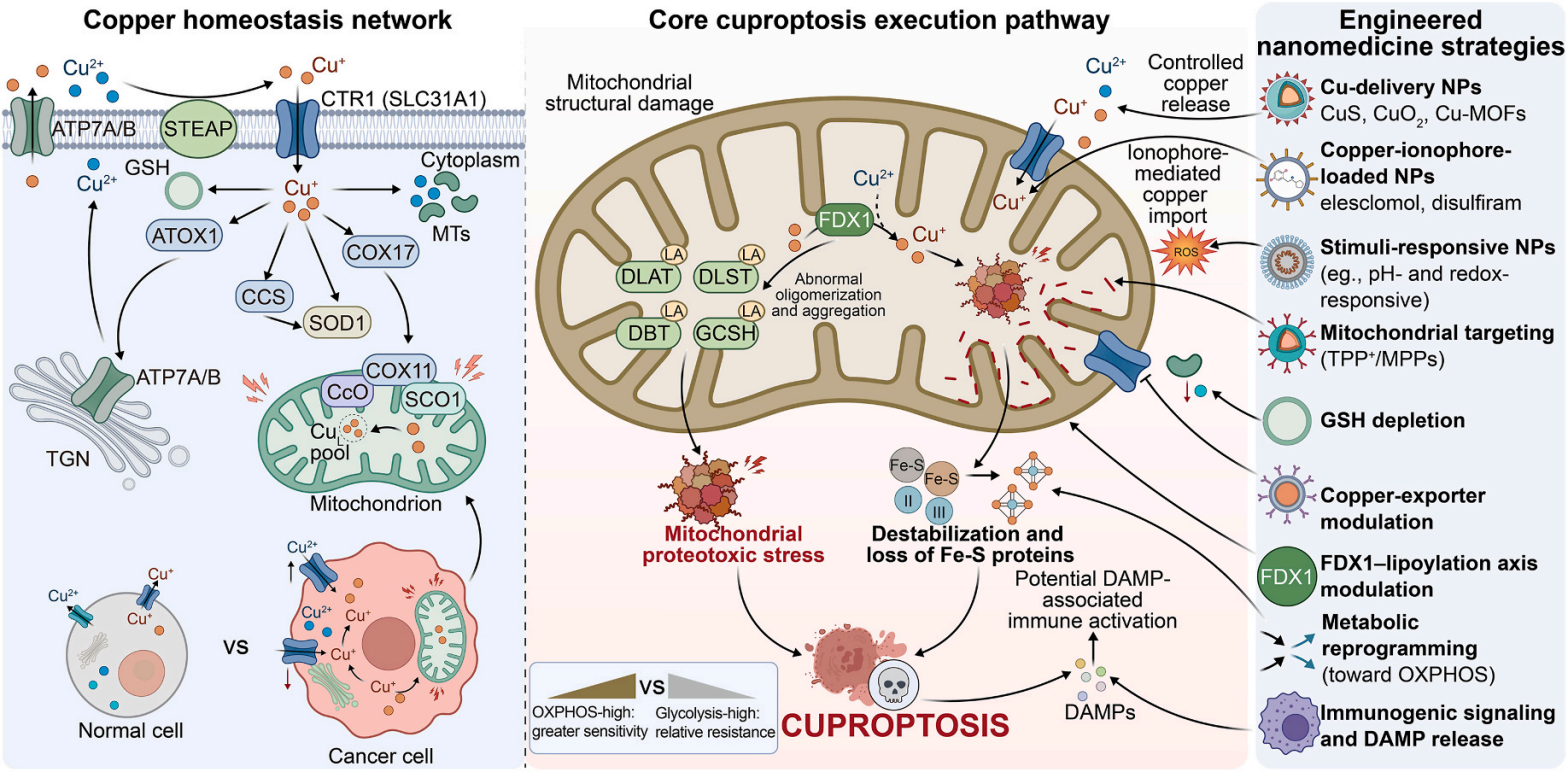
**Copper homeostasis, core cuproptosis execution, and nanomedicine engineering strategies in cancer.** Cellular copper homeostasis is regulated through uptake, intracellular trafficking, buffering, compartmentalization, and export. Extracellular Cu^2+^ is reduced to Cu ^+^ by STEAP proteins and taken up mainly through CTR1, encoded by *SLC31A1*. ATOX1 delivers copper to ATP7A/ATP7B, CCS supplies copper to SOD1, and COX17-dependent pathways support cytochrome *c* oxidase assembly. Glutathione, metallothioneins, and ATP7A/ATP7B limit labile intracellular copper. Cuproptosis occurs when excess mitochondrial copper binds lipoylated tricarboxylic acid-cycle proteins, particularly DLAT, causing protein oligomerization, Fe–S protein loss, mitochondrial proteotoxic stress, and bioenergetic failure. FDX1 supports protein lipoylation and has been proposed to facilitate copper reduction. OXPHOS-high tumor cells are generally more sensitive, whereas glycolysis-high cells are relatively resistant. Cuproptotic injury may also promote DAMP release and immune activation. Nanomedicine strategies include copper-delivery nanoparticles, copper-ionophore-loaded nanoparticles, stimulus-responsive copper release, mitochondrial targeting, GSH depletion, copper-exporter modulation, modulation of the FDX1–lipoylation axis, metabolic reprogramming toward OXPHOS, and immunogenic signaling. **Note:** Cu, copper; Cu^+^, cuprous copper; Cu^2+^, cupric copper; STEAP, six-transmembrane epithelial antigen of the prostate; CTR1, copper transporter 1; SLC31A1, solute carrier family 31 member 1; ATOX1, antioxidant 1 copper chaperone; CCS, copper chaperone for superoxide dismutase; COX17, cytochrome *c* oxidase copper chaperone 17; ATP7A, ATPase copper transporting alpha; ATP7B, ATPase copper transporting beta; CcO, cytochrome c oxidase; COX11, cytochrome *c* oxidase copper chaperone COX11; SOD1, superoxide dismutase 1; SCO1, synthesis of cytochrome *c* oxidase 1; TGN, trans-Golgi network; FDX1, ferredoxin 1; DLAT, dihydrolipoamide S-acetyltransferase; DLST, dihydrolipoamide S-succinyltransferase; DBT, dihydrolipoamide branched-chain transacylase E2; GCSH, glycine cleavage system protein H; LA, lipoyl group; Fe–S, iron–sulfur; ROS, reactive oxygen species; GSH, glutathione; MTs, metallothioneins; OXPHOS, oxidative phosphorylation; DAMPs, damage-associated molecular patterns; NPs, nanoparticles; CuS, copper sulfide; CuO, copper oxide; Cu-MOFs, copper-based metal–organic frameworks; TPP^+^, triphenylphosphonium; MPPs, mitochondria-penetrating peptides.

Cuproptosis is not a diffuse consequence of metal overload. It arises when mitochondrial copper stress coincides with an intact lipoylation pathway and sufficient dependence on oxidative metabolism [10,12,36]. For therapeutic design, total cellular copper uptake is less informative than its subcellular fate. The efficacy of a cuproptosis-based strategy depends less on bulk copper uptake than on efficient mitochondrial delivery under biochemical conditions that preserve the susceptibility of lipoylated enzymes. A mechanistically grounded framework should first consider copper homeostasis, then delineate the core execution pathway, and finally identify the tumor states that permit effective engagement of cuproptosis (Fig. 1) [10,33,37,38].

### 2.1 Copper homeostasis in mammalian cells

Copper is essential for mammalian physiology, but its chemical reactivity requires tight control. Its ability to cycle between Cu^+^ and Cu^2+^ allows copper to act as a cofactor for enzymes involved in mitochondrial respiration, antioxidant defense, connective tissue remodeling, catecholamine synthesis, and iron mobilization [[1], [2], [3],^39^]. At the same time, this redox flexibility allows excess copper to disturb thiol homeostasis, drive damaging redox reactions, and displace other metals from susceptible metalloproteins [2,40]. Mammalian cells do not maintain a freely diffusible pool of copper. Rather, intracellular copper availability is tightly regulated through coordinated uptake, trafficking, buffering, compartmentalization, and export. This homeostatic network not only limits toxicity but also determines where copper is biologically available and which subcellular compartments first experience stress when buffering capacity is exceeded [33,[41], [42], [43]]. Understanding this network is central to cuproptosis biology because it determines whether therapeutic copper reaches mitochondria and engages lipoylated substrates before it is buffered or exported.

At the level of cellular entry and intracellular routing, copper uptake is mediated mainly by CTR1, encoded by *SLC31A1*, which functions as a high-affinity transporter for Cu^+^ [2,3,44]. Extracellular Cu^2+^ is usually reduced before uptake, allowing CTR1 to import the cuprous form [45]. Once inside the cell, copper is distributed through a dedicated chaperone network rather than by passive diffusion. Antioxidant 1 copper chaperone (ATOX1) transfers copper to the trans-Golgi network, where ATP7A and ATP7B incorporate it into secretory cuproenzymes and, under conditions of copper excess, can relocalize to support sequestration or efflux [1,17,[46], [47], [48]]. Copper chaperone for superoxide dismutase (CCS) delivers copper to superoxide dismutase 1 (SOD1), whereas cytochrome *c* oxidase copper chaperone COX17 and related proteins direct copper to cytochrome *c* oxidase [1,39,49,50]. This routing system is also relevant to cancer therapy because it defines the kinetic and spatial constraints that therapeutic copper must overcome. Copper delivery alone is unlikely to be sufficient because exogenous copper can be rapidly buffered, sequestered or exported before reaching mitochondria [33,43,51,52].

Beyond trafficking, cells also limit copper toxicity through buffering systems centered on glutathione and metallothioneins. Glutathione is a major intracellular copper-binding metabolite and contributes to the early phases of copper handling after CTR1-mediated uptake [40,53,54]. Metallothioneins provide an additional layer of sequestration through cysteine-rich metal-binding motifs [3,55]. Together, these buffering systems help define the threshold at which copper becomes toxic. Cuproptosis is likely to occur only when copper influx and mitochondrial copper delivery exceed the combined buffering capacity of glutathione, metallothioneins, and copper efflux pathways. This consideration is especially relevant in cancer, where thiol metabolism and metal detoxification programs are often transcriptionally reinforced [33,38,42]. Copper homeostasis thus determines both cellular tolerance and whether copper remains available long enough to engage the cuproptosis pathway.

These homeostatic principles are closely connected to tumor biology. Elevated copper levels have been reported across multiple malignancies, and copper can support oncogenic signaling, autophagy-dependent tumor maintenance, oxidative metabolism, and metastatic progression [5,6,56,57]. Brady et al. showed that copper is required for oncogenic BRAF signaling through MEK1 [8]. Tsang et al. later demonstrated that copper directly regulates unc-51-like kinase 1/2 (ULK1/2), linking copper availability to autophagy and lung adenocarcinoma growth [7]. Ishida et al. found that bioavailable copper supports oxidative phosphorylation and tumor expansion. In contrast, Ramchandani et al. showed that systemic copper depletion impairs mitochondrial metabolism and suppresses metastasis in triple-negative breast cancer [58,59]. Together, these studies suggest that many tumors do not accumulate copper passively, but instead rely on copper-dependent signaling and bioenergetic pathways. This dependence is therapeutically important because the same copper requirement that supports tumor growth may also create vulnerability when copper is redirected toward mitochondrial proteotoxic stress. However, copper dependence alone does not define cuproptosis, because cell death still requires a specific mitochondrial substrate context and execution pathway.

### 2.2 Core execution pathway

The molecular identity of copper-induced cell death remained undefined until Tsvetkov et al. showed in 2022 that copper ionophore-induced killing depends on mitochondrial protein lipoylation and Fe–S biology rather than on canonical apoptotic or necroptotic machinery [10]. Genome-wide CRISPR screens linked resistance to elesclomol–copper to loss of FDX1 and genes required for protein lipoylation, including lipoyl synthase (LIAS), lipoyltransferase 1 (LIPT1), and dihydrolipoamide dehydrogenase (DLD) [10,36]. These findings established cuproptosis as a selective mitochondrial stress pathway with a narrow and biochemically defined substrate basis [10,37].

FDX1 is a central regulator of this pathway. It functions as a mitochondrial ferredoxin and was the strongest genetic determinant of cuproptosis identified in the original screen [10]. FDX1 has been proposed to reduce ionophore-bound Cu^2+^ to Cu^+^, promoting copper release within mitochondria [10,17]. FDX1 is also required for efficient protein lipoylation. Dreishpoon et al. later showed that FDX1 directly binds LIAS and promotes its interaction with the lipoyl carrier machinery, supporting a direct LIAS-dependent role in protein lipoylation rather than an indirect effect through global Fe–S control alone [60]. Taken together, these findings place FDX1 at the intersection of copper activation and maintenance of the lipoylated substrates that define cuproptotic vulnerability [10,60].

The main downstream targets of cuproptosis are lipoylated mitochondrial proteins. In mammalian cells, lipoylation is restricted to a small group of enzymes, including dihydrolipoamide S-acetyltransferase (DLAT), dihydrolipoamide S-succinyltransferase (DLST), dihydrolipoamide branched-chain transacylase E2 (DBT), and glycine cleavage system protein H (GCSH) [10,61]. Under normal conditions, these proteins support acyl-group transfer within multienzyme complexes required for mitochondrial carbon metabolism. Under copper overload, copper binds lipoyl groups and drives abnormal oligomerization and aggregation, particularly of DLAT [10,12]. Cuproptosis, therefore, does not reflect diffuse mitochondrial poisoning. Instead, it involves a narrow group of lipoylated enzymes that are both metabolically important and structurally vulnerable to copper-dependent aggregation. The consequence is not only loss of enzymatic function but also the generation of mitochondrial proteotoxic stress [10,60].

Another key component of the pathway is the destabilization of Fe–S cluster proteins [10]. Fe–S clusters are required for respiratory chain activity, aconitase function, and multiple mitochondrial metabolic processes [16,62]. Copper stress reduces the abundance of Fe–S proteins in cuproptosis-permissive cells, although the precise biochemical mechanism remains under investigation [10,16,62,63]. Lipoylated protein aggregation and Fe–S protein loss occur together during cuproptosis, although their causal relationship remains unresolved. Aggregation increases proteotoxic stress within the mitochondrial matrix, whereas Fe–S loss impairs electron transfer and metabolic functions needed to maintain organelle integrity. Cuproptosis can therefore be understood as a coupled failure of mitochondrial proteostasis and bioenergetic function [10,33,38].

This execution logic also helps explain the metabolic selectivity of cuproptosis. Cells dependent on oxidative phosphorylation are markedly more sensitive to elesclomol–copper than cells in a glycolytic state [10]. Although this pattern is often described simply as dependence on mitochondrial metabolism, it more specifically reflects sustained flux through lipoylated mitochondrial enzyme systems and continued reliance on oxidative carbon metabolism. By contrast, strongly glycolytic cells are relatively protected because they rely less on lipoylated mitochondrial enzyme complexes and can better tolerate impaired oxidative metabolism. Cuproptosis is therefore more closely linked to oxidative metabolic commitment than to copper accumulation alone [10].

For nanomedicine studies, it is particularly important to distinguish bona fide cuproptosis from broader copper-induced cytotoxicity. Convincing evidence of cuproptosis should combine copper dependence with pathway-specific validation. Core evidence includes dependence on FDX1 or the protein-lipoylation machinery, aggregation of lipoylated proteins, and loss of Fe–S proteins. Copper-chelator rescue and exclusion of dominant alternative death pathways provide additional support [10,12,16,36,60,64]. Therefore, copper accumulation, reactive oxygen species (ROS) production, mitochondrial dysfunction, reduced cell viability, or tumor growth inhibition should not be interpreted as definitive evidence of canonical cuproptosis unless pathway-specific markers and rescue experiments are included. This distinction is especially relevant to cancer nanomedicine, where copper-based platforms may intentionally combine cuproptosis with other death programs. For example, a recently developed copper-based nanoinducer was reported to disrupt intracellular copper and redox homeostasis, inducing cuproptosis/disulfidptosis-associated tumor killing and antitumor immune activation [64]. This example highlights the therapeutic potential of metal-ion-based cancer nanomedicine, while also reinforcing the need to define mechanism-specific endpoints when attributing tumor suppression to cuproptosis.

Within the original discovery framework, cuproptosis is mechanistically distinct from apoptosis, ferroptosis, and necroptosis [10,13]. At the same time, copper-induced mitochondrial injury unfolds in a broader cellular context in which redox stress, organelle quality control, innate immune sensing, and other death-related pathways remain active. As a result, cuproptosis may retain a distinct execution logic while also contributing to wider stress responses that shape tumor cell killing, inflammatory output, and treatment response [10,38,65]. These mechanistic requirements also help explain why cuproptosis sensitivity varies so markedly across tumor cells and tumor states.

### 2.3 Determinants of cancer cell susceptibility

Tumor sensitivity to cuproptosis is highly heterogeneous and is better understood as a cellular state rather than a single marker. A cuproptosis-permissive tumor cell is one in which oxidative dependence, copper accessibility, lipoylation competence, and limited detoxification capacity coincide. This framework helps explain why vulnerability is highly context-dependent, with some tumors remaining sensitive whereas others evade copper-induced death despite substantial copper exposure [10,33,38].

A major determinant of sensitivity is metabolic phenotype. Cells with high oxidative phosphorylation and active TCA-cycle flux are generally vulnerable, whereas glycolysis-dominant cells are relatively protected [10]. In tumors, however, this distinction is complicated by metabolic heterogeneity and plasticity. Solid tumors often contain subpopulations with different degrees of mitochondrial dependence, and therapeutic pressure can drive shifts between glycolytic and oxidative states. Cuproptosis sensitivity is therefore dynamic rather than fixed. Tumors that appear resistant at baseline may become vulnerable after metabolic rewiring, whereas initially responsive tumors may escape by shifting toward glycolysis or reducing exposure of lipoylated mitochondrial substrates [10,33,38].

Copper accessibility depends on uptake, intracellular routing, buffering and efflux, which together determine whether bioavailable copper reaches mitochondria. High CTR1 expression favors copper uptake, whereas ATP7A and ATP7B reduce effective copper stress by promoting sequestration or export [44,47]. These proteins therefore influence not only total copper flux but also whether copper remains bioavailable long enough to reach mitochondria in a toxic form. Their relevance is further supported by the overlap between copper handling and platinum pharmacology. CTR1 contributes to cisplatin accumulation, whereas ATP7A and ATP7B are associated with platinum resistance [66,67]. This relationship suggests that tumors with a transport profile linked to platinum resistance may also require stronger strategies to sustain cuproptotic copper stress [33,[68], [69], [70]]. Beyond transport, antioxidant and metal-buffering capacity further shape susceptibility. Glutathione is a major intracellular copper buffer, and metallothioneins further increase cellular tolerance to copper [40,[53], [54], [55]]. This buffering axis is especially relevant in treatment-resistant tumors, where nuclear factor erythroid 2-related factor 2 (NRF2)-linked antioxidant programs, thiol metabolism, and metal detoxification are often reinforced. A tumor may therefore remain resistant to cuproptosis even when copper levels are high if labile copper can be neutralized before sustained mitochondrial delivery is achieved [38,42,71]. In this sense, copper exposure alone is not a sufficient predictor of sensitivity unless it is interpreted together with the transport and detoxification systems that regulate copper persistence.

The integrity of the lipoylation machinery is another major determinant of susceptibility. FDX1 is especially important because loss of FDX1 or reduced FDX1 activity can impair cuproptosis even when cellular copper exposure is adequate [10,60]. Recent studies also show that tumor cells can acquire resistance through suppression of the FDX1 axis. Sun et al. reported that AKT1 phosphorylates FDX1 and promotes cuproptosis resistance in triple-negative breast cancer [72]. Yang et al. showed that HIF-1α drives resistance under hypoxic conditions by suppressing the lipoylation-dependent substrate state and reinforcing copper-sequestering programs [38]. Together, these findings indicate that cuproptosis is not determined by copper exposure alone. Tumor cells can also resist death by disrupting the mitochondrial substrate architecture required for pathway execution. Susceptibility therefore depends not only on copper availability, but also on whether the mitochondrial substrate state required for cuproptotic execution remains intact.

Susceptibility is further influenced by how copper stress intersects with other stress pathways and by the downstream consequences of mitochondrial injury. Interactions between copper overload, iron metabolism, redox imbalance, and ferroptotic permissiveness may all shape the overall response to treatment [[73], [74], [75]]. Mitochondrial injury can also lead to mitochondrial DNA (mtDNA) leakage and activation of cyclic GMP–AMP synthase–stimulator of interferon genes (cGAS–STING) signaling [76,77]. Direct evidence indicates that cuproptosis can engage this pathway, and cuproptotic nanoinducers have also been shown to promote mtDNA-dependent cGAS–STING activation and improve the response to cancer immunotherapy [65,78]. These findings suggest that cuproptosis may contribute not only to mitochondrial cell death but also to innate immune activation. This extension is particularly relevant to nanomedicine, where therapeutic efficacy may depend not only on direct tumor killing, but also on the immunological consequences of mitochondrial copper stress. Because susceptibility depends on copper handling, mitochondrial access, and metabolic state, cuproptosis is amenable to multilevel control by nanomedicine.

### 2.4 Opportunities for nanomedical engineering

The molecular features of cuproptosis create several opportunities for nanomedical engineering. Unlike pathways driven by a single receptor or mutation, cuproptosis depends on a spatially organized vulnerability network involving copper transport, intracellular buffering, mitochondrial access, lipoylated substrate availability, oxidative metabolism, and immune consequences. Nanomedicine is particularly relevant in this setting because it can coordinate these variables as a biological sequence rather than act through copper delivery alone [19,33,78,79].

A central opportunity is to convert copper exposure into tumor-selective mitochondrial copper stress. Total intracellular copper accumulation alone is not a sufficient pharmacodynamic endpoint. The critical requirement is bioavailable copper that reaches mitochondria and persists long enough to engage lipoylated TCA-cycle proteins before being neutralized by glutathione, metallothioneins, or copper efflux pathways [10,33,38]. Mitochondria-directed delivery may improve pathway engagement when cytosolic buffering or misrouting limits copper access to lipoylated substrates. Targeting motifs that favor copper release near or within mitochondria may improve cuproptotic efficacy while reducing the total copper burden required for treatment [10,78,79]. For example, ROS-responsive nanoparticles co-encapsulating elesclomol and copper enhanced cuproptosis and improved the response to anti-programmed death-ligand 1 (anti-PD-L1) therapy, illustrating how tumor-triggered activation and mitochondrial copper delivery can be integrated into one platform [19]. In this sense, effective cuproptosis nanomedicine should be designed around tumor localization, mitochondrial access, and intracellular copper persistence, rather than drug loading alone.

A second opportunity lies in vulnerability amplification. Glycolytic tumor cells are relatively resistant to cuproptosis because they rely less on oxidative carbon metabolism and contain fewer functionally exposed lipoylated mitochondrial substrates [10]. Nanoplatforms may help reshape this context by co-delivering glycolysis inhibitors, mitochondrial metabolic modulators, redox-active agents, or buffering-disruptive components that shift tumor cells toward a more cuproptosis-permissive state [10,33]. This approach is conceptually important because the nanoplatform does not merely transport a copper payload; it also modifies the metabolic and redox conditions that determine whether copper becomes lethal.

Immune coupling provides another important engineering opportunity. Cuproptosis-associated mitochondrial injury can promote immunogenic outputs, including damage-associated molecular pattern (DAMP) release and mtDNA-dependent innate immune signaling [19,78,79]. Cuproptosis-inducing nanoparticles have been reported to reprogram the tumor microenvironment and enhance anti-PD-L1 therapy [19,80]. Cuproptotic nanoinducers can also activate the mtDNA–cGAS–STING axis, and inhalable nanodevices for lung metastasis immunotherapy have further combined enhanced cuproptosis with cGAS–STING activation and PD-L1 blockade [78,79]. These findings suggest that cuproptosis nanomedicine may function not only as a tumor-cell killing strategy, but also as a platform for converting mitochondrial copper stress into antitumor immune engagement.

These opportunities should not be interpreted as justification for indiscriminate multifunctional design. Cuproptosis intersects with ferroptotic permissiveness, inflammatory signaling, redox imbalance, and organelle stress responses [[73], [74], [75], [76],^81^,^82^]. However, each added module should address a defined biological barrier, such as insufficient mitochondrial copper delivery, excessive copper buffering, glycolytic escape, immune silence, or poor tumor retention. Future platforms are likely to be most effective when copper activation, mitochondrial targeting, buffering depletion, metabolic sensitization, and immune engagement are organized as a coherent therapeutic sequence. Overall, the engineering value of cuproptosis lies in redirecting copper handling toward tumor-selective mitochondrial proteotoxic stress while limiting metabolic escape, microenvironmental resistance, and systemic copper toxicity.

## 3 Design principles of cuproptosis nanomedicine

**Fig. 2:**
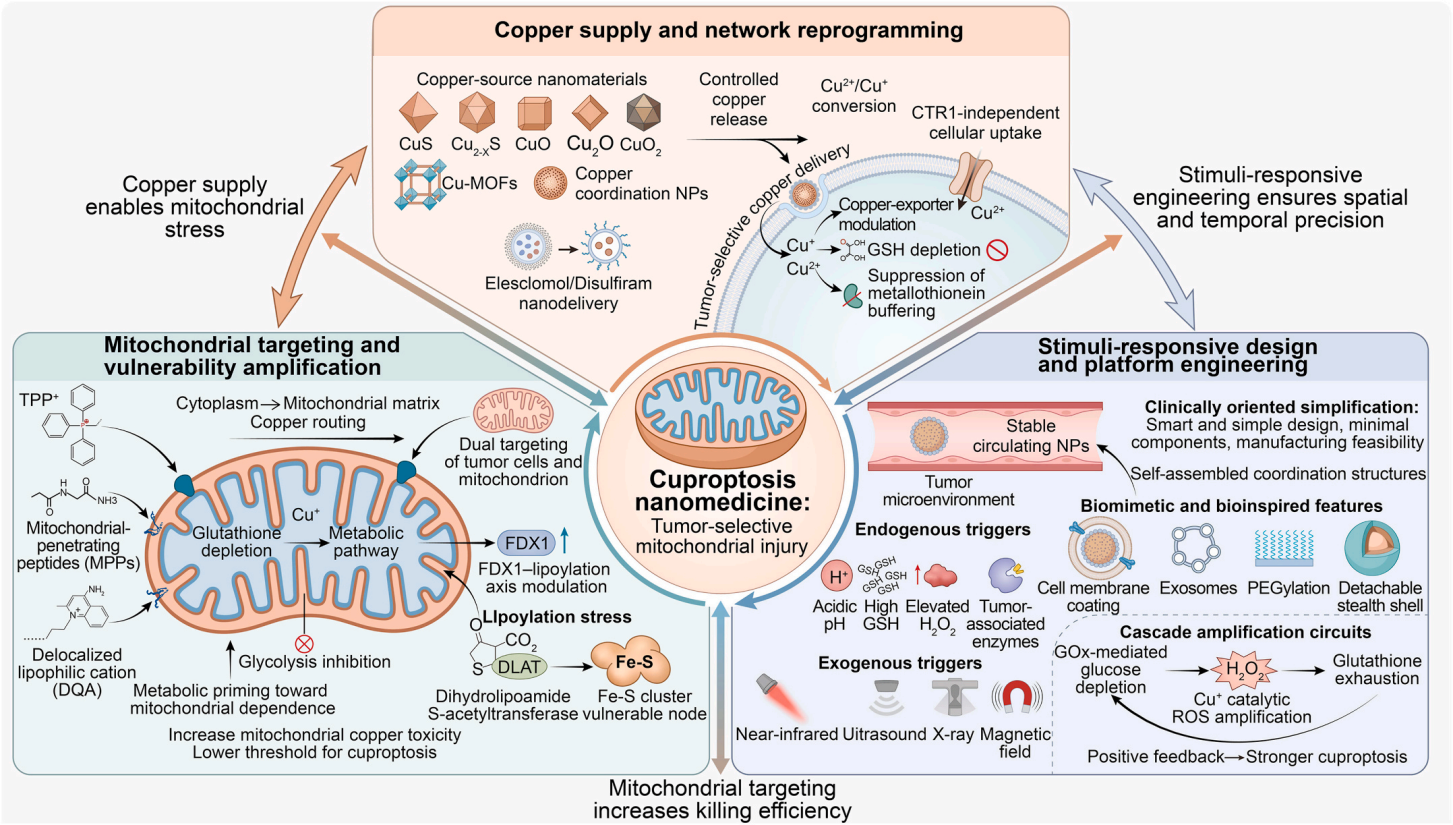
**Mechanism-guided design principles for engineering cuproptosis with nanomedicine.** Cuproptosis-oriented nanoplatforms can be designed through three interconnected strategies. Copper supply and network reprogramming increase intracellular bioavailable copper through copper-source nanomaterials, copper ionophore delivery, controlled copper release, Cu^2+^/Cu^+^ conversion, CTR1-independent cellular uptake, inhibition of ATP7A/ATP7B-mediated copper efflux, glutathione depletion, and suppression of metallothionein buffering. Mitochondrial targeting and vulnerability amplification enhance copper delivery to the mitochondria or mitochondria-proximal compartments through triphenylphosphonium, mitochondria-penetrating peptides, or delocalized lipophilic cations. Glycolysis inhibition, increased reliance on oxidative phosphorylation, and modulation of the FDX1–lipoylation axis further increase tumor-cell susceptibility to cuproptosis. Stimuli-responsive design and platform engineering use endogenous triggers, including acidic pH, elevated glutathione and hydrogen peroxide, and tumor-associated enzymes, together with exogenous triggers, including near-infrared irradiation, ultrasound, X-rays, and magnetic fields, to improve the spatial and temporal control of copper activation. Biomimetic coatings, exosomes, PEGylation, detachable stealth shells, and cascade amplification circuits can further improve circulation, tumor accumulation, intracellular activation, and therapeutic efficacy. Together, these strategies aim to convert copper delivery into tumor-selective mitochondrial proteotoxic stress while limiting systemic toxicity. **Note:** ATP7A/ATP7B, ATPase copper transporting alpha/beta; CTR1, copper transporter 1; Cu, copper; Cu^+^, cuprous copper; Cu^2+^, cupric copper; CuS, copper sulfide; Cu_2-x_S, copper-deficient copper sulfide; CuO, copper oxide; Cu_2_O, cuprous oxide; CuO_2_, copper peroxide; Cu-MOFs, copper-based metal–organic frameworks; DQA, dequalinium; DLAT, dihydrolipoamide S-acetyltransferase; FDX1, ferredoxin 1; Fe–S, iron–sulfur; GOx, glucose oxidase; GSH, glutathione; H_2_O_2_, hydrogen peroxide; MPPs, mitochondria-penetrating peptides; MTs, metallothioneins; NIR, near-infrared; NPs, nanoparticles; OXPHOS, oxidative phosphorylation; PEG, polyethylene glycol; ROS, reactive oxygen species; TME, tumor microenvironment; TPP^+^, triphenylphosphonium.

Cuproptosis is well suited to nanomedical engineering because its vulnerability is spatially organized, biochemically definable, and closely linked to subcellular context. Copper stress alone is unlikely to be sufficient; effective induction depends on whether copper remains bioavailable, reaches mitochondria, encounters tumor cells with a lipoylation-dependent substrate state, and is released within a window that limits buffering adaptation and off-target toxicity [10,24,25]. Cuproptosis-based nanomedicines should be evaluated by their ability to generate tumor-selective mitochondrial copper stress, not simply by copper loading or formulation complexity [29,31]. Cuproptosis-oriented nanomedicines can be organized according to their primary engineering functions, including copper supply, copper oxidation-state control, catalytic amplification, mitochondrial targeting, copper-homeostasis reprogramming, and responsive release. Copper-depleting approaches are discussed separately as inverse copper-disequilibrium strategies because they are mechanistically distinct from copper-overload-driven cuproptosis. Representative copper-modulating strategies are summarized in Fig. 2 and Table 1. A translational comparison of these platforms requires attention to copper loading, copper oxidation state, stimulus-responsive release behavior, serum stability, mitochondrial accumulation, biodistribution, clearance route, and organ toxicity, because these parameters determine whether copper stress can be made both tumor-selective and clinically tolerable. These studies show that cuproptosis nanomedicine is evolving from first-generation copper-overload systems toward second-generation platforms that actively control copper release, subcellular delivery, redox buffering, metabolic vulnerability, and antitumor immunity. Nevertheless, mechanistic rigor and pharmacological characterization vary substantially across studies, and future work should combine pathway-specific validation, including FDX1 dependence, lipoylated DLAT aggregation, Fe–S protein loss, and rescue experiments, with standardized formulation, biodistribution, clearance, and safety data.

**Table 1:** Representative copper-modulating nanomedicine strategies in cancer.

Design category	Representative platforms	Engineering rationale	Cuproptosis-related mechanism	Copper activation/release feature	In vivo translational considerations	Therapeutic advantages	Key limitations	Ref.
Copper sulfide and copper-deficient chalcogenide nanoparticles	CuS and Cu_2-x_S nanoparticles	Serve simultaneously as copper reservoirs and photothermal agents	Acidic or GSH-rich tumor environments promote copper ion release; NIR irradiation accelerates copper liberation and mitochondrial stress	NIR- or TME-enhanced copper release; Cu-deficient chalcogenides also provide photothermal activation	Useful for image-guided or light-controlled therapy; liver/spleen accumulation and limited tissue penetration remain key concerns	Integrates copper delivery, photothermal therapy, and imaging potential; suitable for light-controlled cuproptosis	Limited tissue penetration of NIR light; risk of liver and spleen accumulation; cuproptosis-specific validation often insufficient	[[83], [84], [85]]
Copper oxide and cuprous oxide nanoparticles	CuO and Cu_2_O nanoparticles	Acid-sensitive copper release and intrinsic redox activity	Release Cu^2+^/Cu^+^ under acidic conditions; Cu^2+^ reacts with GSH to generate Cu^+^ and deplete antioxidant buffering	pH-sensitive Cu^2+^/Cu^+^ release; Cu_2_O provides direct cuprous copper and supports GSH-mediated redox cycling	High local redox activity may improve efficacy but increases the need for dissolution control and organ toxicity assessment	Direct copper supply; can amplify CDT and GSH depletion	Potential off-target oxidative toxicity; dissolution kinetics must be carefully controlled	[[86], [87], [88]]
Copper peroxide nanodots	CuO_2_ and Lipo-Ele@CuO_2_	Self-supplying H_2_O_2_ and copper source	Acid-triggered decomposition releases Cu^2+^ and H_2_O_2_; Cu-mediated Fenton-like reactions enhance oxidative stress and may support cuproptosis	Acid-triggered co-release of Cu^2+^ and H_2_O_2_; self-supplied H_2_O_2_ strengthens catalytic oxidative stress	Peroxide chemistry may increase potency but requires careful evaluation of systemic oxidative toxicity and local inflammatory injury	Couples cuproptosis with chemodynamic therapy; overcomes limited endogenous H_2_O_2_	ROS-mediated cytotoxicity may confound cuproptosis attribution; safety of peroxide systems requires careful evaluation	[89,90]
Copper-based MOF and coordination polymer frameworks	HKUST-1, Cu-MOF derivatives, and Cu(I) coordination polymer nanoplatforms	High copper loading and porous or coordination-based drug-loading architecture	Framework degradation releases copper ions; co-loaded drugs or enzymes modulate redox status, metabolism or immunity	Framework degradation enables stimulus-responsive copper release; Cu(I) coordination systems can provide a cuproptosis-relevant copper state	High loading and modularity are attractive, but framework stability, metal leakage, and batch reproducibility need systematic pharmacological evaluation	Flexible co-delivery of photosensitizers, chemotherapeutics, immune agonists or metabolic regulators	MOF stability, biodegradation, metal leakage and batch reproducibility remain major concerns	[[91], [92], [93], [94]]
Hollow metal-based and bimetallic nano-amplifiers	Copper-dithiocarbamate/artemisinin hollow nanoplatforms and hollow CaCO_3_/Cu coordination polymer amplifiers	Use hollow architectures to increase internal loading, enable TME-responsive degradation, and coordinate metal-ion release with organelle-stress amplification	Released Cu^2+^ promotes DLAT oligomerization and mitochondrial dysfunction; co-loaded or in situ generated modules amplify oxidative stress, ER stress, Ca^2+^ redistribution, ferroptosis, paraptosis, or apoptosis	Hollow structure-enabled TME-responsive release; Cu^2+^ liberation can be coupled with artemisinin activation, GSH depletion, Ca^2+^ redistribution, and dithiocarbamate–copper complex formation	Promising for synergistic therapy, but hollow-structure reproducibility, bimetallic composition control, metal leakage, biodistribution, and long-term organ safety require systematic evaluation	High co-loading capacity; strong cascade amplification; integrates cuproptosis with ferroptosis, paraptosis, apoptosis, and organelle-stress therapy	Complex synthesis; difficult quality control; multiple death pathways may complicate attribution to canonical cuproptosis	[95,96]
Copper single-atom nanozymes	Cu single-atom catalysts on carbon, nitrogen-doped supports or related matrices, and Co–N–C single-atom nanozyme delivering copper	Maximize catalytic activity or metabolic regulation per active site while lowering total metal burden	Peroxidase-like, glutathione oxidase-like or redox catalytic activities amplify copper stress; metabolic reprogramming may increase cuproptosis susceptibility	Atomically dispersed catalytic copper sites or copper-delivering single-atom systems amplify redox reactions at low total metal dose	Lower metal burden may improve safety, but long-term fate, degradation, and clearance of catalytic sites remain insufficiently defined	Atom-efficient; may reduce total copper burden; strong catalytic amplification	Complex synthesis and characterization; long-term fate of catalytic sites remains unclear	[[97], [98], [99]]
Copper–polyphenol coordination networks	Cu–tannic acid, Cu–gallic acid, Cu–polyphenol assemblies, and atovaquone-coordinated Cu-polyphenol nanoplatforms	Mild coordination assembly with pH-responsive degradation and tunable ligand function	Tumor acidity disrupts coordination bonds and releases copper; polyphenol ligands may add redox, mitochondrial or immune-modulatory effects	pH-responsive coordination-bond dissociation; ligand composition can tune copper release and mitochondrial or immune effects	Biocompatible assembly is favorable, but composition heterogeneity and environment-dependent release profiles need tighter standardization	Simple preparation; good biocompatibility; suitable for responsive delivery	Variable composition; weak structural precision; release profiles may differ across biological environments	[[100], [101], [102]]
Protein-templated and biomineralized copper nanocarriers	Albumin, ferritin or protein-templated copper nanoparticles, and ferritin-CuS nanocages	Use endogenous protein scaffolds to improve circulation, biocompatibility and tumor accumulation	Protein carrier improves tumor accumulation; copper release induces mitochondrial proteotoxic stress or cuproptosis-associated redox injury	Protein-confined copper loading with biologically mediated release or degradation; scaffold may reduce premature copper exposure	Protein carriers may improve biocompatibility and manufacturability, but copper loading heterogeneity and protein denaturation should be monitored	Better biocompatibility and manufacturability than many inorganic platforms	Copper loading heterogeneity; potential immunogenicity or protein denaturation during preparation	[[103], [104], [105]]
Nanoformulated copper ionophores	Elesclomol- or DSF/Cu-loaded liposomes, PLGA nanoparticles, albumin nanoparticles, lipid nanoparticles, and Cel-Cu nanoparticles	Improve solubility, stability, pharmacokinetics and tumor accumulation of classical ionophores	Elesclomol transports copper to mitochondria; DSF/Cu complexes increase intracellular copper stress; Cel-Cu NPs combine copper-ionophore activity with GSH depletion to amplify DLAT-associated cuproptosis	Carrier-stabilized copper shuttling; tumor-site or redox-responsive activation can improve ionophore delivery and intracellular copper transport	Improves drug stability and tumor retention, but systemic ionophore toxicity, plasma instability, and patient selection remain critical	Strong mechanistic precedent; repurposing potential; easier pharmacological interpretation	Elesclomol and DSF have systemic toxicity and stability issues; patient selection is essential	[19,86,[106], [107], [108], [109], [110], [111]]
Stimulus-responsive copper-complex nanoparticles	ROS-responsive Cu(I) nanoparticles	Stabilize a chemically defined Cu(I) complex and restrict copper activation to oxidative tumor environments	ROS-triggered Cu(I) release increases mitochondrial copper accumulation, DLAT oligomerization, and mitochondrial proteotoxic stress	Thioketal cleavage enables H_2_O_2_-responsive Cu(I) release; approximately 91.4% release with H_2_O_2_ versus 11.5% in PBS after 36 h	Provides direct delivery of a cuproptosis-relevant copper state, but ROS heterogeneity, premature oxidation, polymer degradation, and long-term safety require evaluation	Defined copper oxidation state; stimulus-responsive activation; immune engagement	Tumor ROS levels are heterogeneous; release specificity and systemic stability require further validation	[112]
Mitochondria-targeted copper nanomedicines	Systems modified with TPP^+^, dequalinium, or mitochondria-penetrating peptides; pyruvate-Cu nanoparticles	Enhanced copper delivery to mitochondria, where lipoylated targets are located	Increases mitochondrial copper exposure and may promote DLAT/DLST aggregation and Fe–S protein loss	Mitochondria-directed copper transport; targeting may depend on ΔΨm, pyruvate metabolism, lipophilic cations, or mitochondria-penetrating motifs	Subcellular targeting can lower required copper dose, but mitochondrial toxicity in normal OXPHOS-dependent tissues should be evaluated	Enhances cuproptosis specificity per unit copper dose; may widen therapeutic window	ΔΨm-dependent targeting may fail in damaged or heterogeneous tumors; mitochondrial toxicity to normal tissues must be assessed	[[113], [114], [115], [116]]
Copper homeostasis-reprogramming nanomedicines	Copper delivery combined with ATP7A-targeting or broader copper-exporter regulation, GSH depletion, metallothionein modulation, or transporter-directed nanoregulation	Disrupt copper import–efflux–buffering balance rather than simply increasing copper dose	Enhances intracellular free copper by suppressing efflux and buffering systems	Copper retention strategy through exporter inhibition, buffering depletion, or transporter/homeostasis regulation	Mechanistically strong for resistant tumors, but multi-component design increases manufacturing and regulatory complexity	Mechanistically sophisticated; may overcome adaptive resistance	Multi-component systems may be difficult to manufacture and regulate	[88,102,[117], [118], [119]]
Inverse copper-disequilibrium strategies	ZnS-based copper nanoconsumers, copper-chelating nanoparticles, mitochondria-targeted copper-depleting nanoparticles, and tetrathiomolybdate-related copper-depletion approaches	Remove or sequester bioavailable copper rather than increase copper loading; should be discussed separately from copper-supplying platforms	Suppress copper-dependent tumor biology by impairing mitochondrial respiration, copper enzymes, angiogenesis, extracellular matrix remodeling, or metastatic niche formation; mechanistically distinct from copper-overload-driven FDX1–DLAT cuproptosis	Copper sequestration, copper consumption, or systemic copper depletion rather than copper release; may reduce bioavailable copper pools, mitochondrial copper availability, or copper-dependent microenvironmental remodeling	May reduce copper-overload toxicity and has translational relevance, but systemic copper deficiency, organ-specific copper depletion, and mechanism-specific attribution require careful evaluation	Useful for copper-addicted tumors; provides a comparative or inverse strategy for copper-disequilibrium therapy, metastasis control, and microenvironment modulation	Not a conventional copper-supply strategy; should not be described as canonical cuproptosis unless FDX1 dependence, lipoylated DLAT aggregation, Fe–S protein loss, and rescue evidence are demonstrated	[[120], [121], [122], [123]]

Note: Cu, copper; CuS, copper sulfide; Cu_2-x_S, copper-deficient copper sulfide; CuO, copper oxide; Cu_2_O, cuprous oxide; CuO_2_, copper peroxide; Lipo-Ele@CuO_2_, liposome-encapsulated elesclomol and copper peroxide nanocomposite; H_2_O_2_, hydrogen peroxide; GSH, glutathione; TME, tumor microenvironment; NIR, near-infrared; ROS, reactive oxygen species; CDT, chemodynamic therapy; MOF, metal–organic framework; HKUST-1, Hong Kong University of Science and Technology-1 copper benzene-1,3,5-tricarboxylate framework; Cu-MOF, copper-based metal–organic framework; Cu(I), cuprous copper; DSF, disulfiram; PLGA, poly(lactic-co-glycolic acid); TPP^+^, triphenylphosphonium; DLAT, dihydrolipoamide S-acetyltransferase; DLST, dihydrolipoamide S-succinyltransferase; Fe–S, iron–sulfur; ATP7A, ATPase copper transporting alpha; ATP7B, ATPase copper transporting beta; siRNA, small interfering RNA; ZnS, zinc sulfide; ΔΨm, mitochondrial membrane potential; FDX1, ferredoxin 1; ER, endoplasmic reticulum; PTT, photothermal therapy; OXPHOS, oxidative phosphorylation; PBS, phosphate-buffered saline; CaCO_3_, calcium carbonate; Ca^2+^, calcium ion.

### 3.1 Copper supply and network reprogramming

A key design challenge is to ensure that delivered copper remains bioavailable and reaches mitochondria before it is neutralized by intracellular defense systems. Copper supply cannot be equated with material loading alone. Whether copper acts as a cuproptotic trigger depends on its chemical form, route of entry, release kinetics, and the extent to which efflux and buffering pathways are disrupted. Accordingly, sustained mitochondrial copper stress generally requires copper delivery to be coordinated with reprogramming of the endogenous copper network.

#### 3.1.1 Copper-source and catalytic nanomaterials

**Fig. 3:**
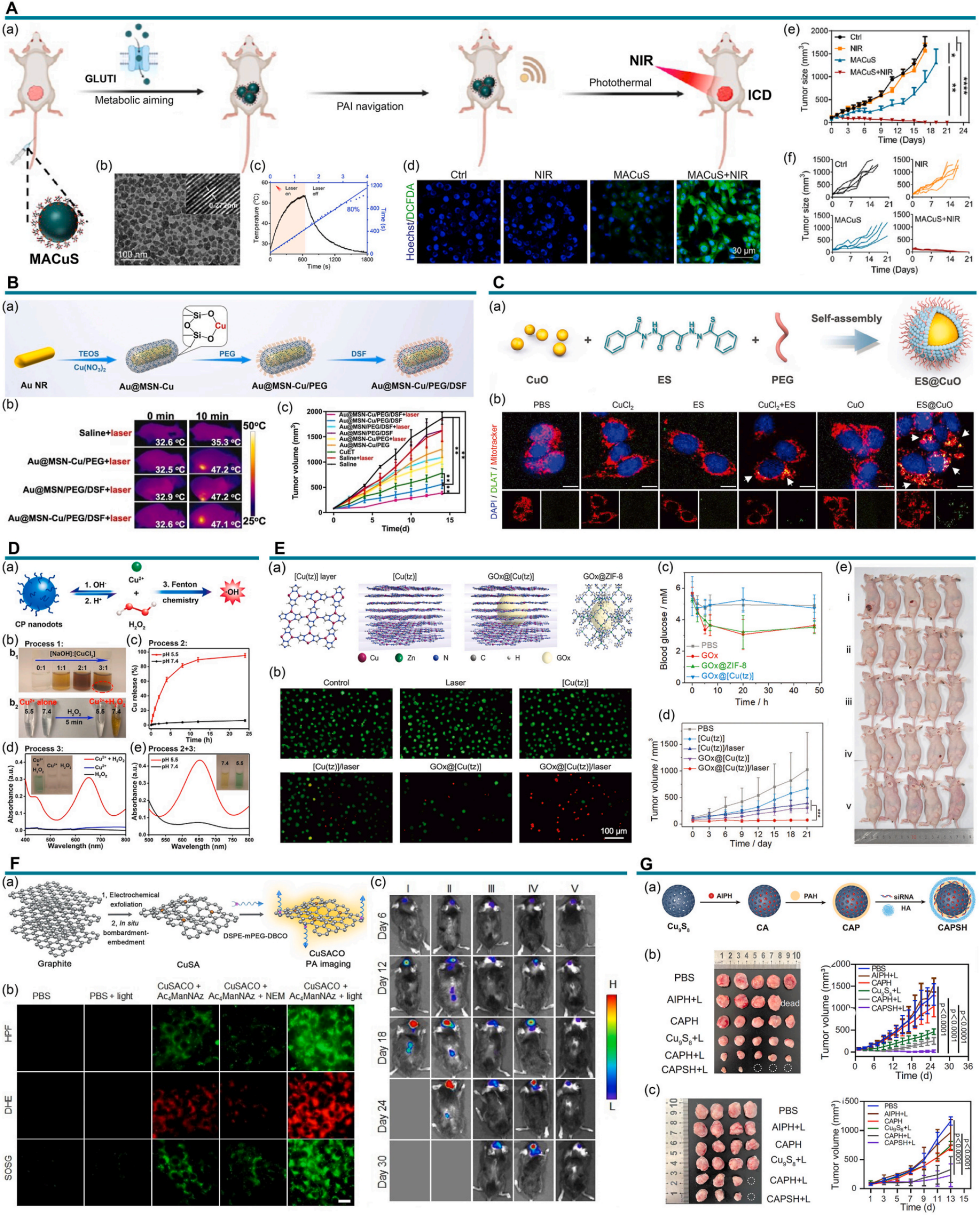
**Copper supply and copper-network reprogramming strategies in cuproptosis-oriented nanomedicine.** (**A**) MACuS enables GLUT1-mediated tumor targeting, PAI-guided NIR photothermal activation, cuproptosis, ICD, and tumor inhibition. (a) Schematic of MACuS tumor targeting, PAI navigation, NIR activation, and ICD. (b) TEM image of MACuS. (c) Photothermal heating curve. (d) ROS staining. (e, f) Average and individual tumor growth curves. Reproduced with permission [83]. Copyright 2025, American Chemical Society. (**B**) Au@MSN-Cu/PEG/DSF achieves NIR-triggered copper and disulfiram release, in situ CuET formation, photothermal therapy, and cuproptosis-promoted tumor suppression. (a) Construction scheme of Au@MSN-Cu/PEG/DSF. (b) Infrared thermal images after laser irradiation. (c) Tumor growth curves. Reproduced with permission [85]. Copyright 2023, Wiley. (**C**) ES@CuO combines CuO-mediated copper supply with elesclomol-assisted mitochondrial copper transport to induce DLAT aggregation. (a) Self-assembly scheme of ES@CuO. (b) Confocal images of DLAT aggregation and mitochondrial localization. Reproduced from Ref. [86] under a Creative Commons license. (**D**) CuO_2_ nanodots self-supply copper and H_2_O_2_ for Fenton-type ROS generation. (a) Schematic of CuO_2_ decomposition and ·OH generation. (b) Photographs of CuO_2_ formation. (c) Cu^2+^ release profile. (d, e) UV–vis spectra showing pH-dependent reaction behavior. Reproduced with permission [90]. Copyright 2019, American Chemical Society. (**E**) GOx@[Cu(tz)] couples GSH-responsive Cu(I) coordination polymer activation with glucose depletion and cuproptosis-based tumor inhibition. (a) Structural schematic of [Cu(tz)], GOx@[Cu(tz)], and GOx@ZIF-8. (b)Live/dead staining images of 5637 cells. (c) Blood glucose changes. (d) Tumor growth curves. (e) Photographs of mice bearing tumors after treatments with PBS (i), [Cu(tz)] (ii), [Cu(tz)]/laser (iii), GOx@[Cu(tz)] (iv), and GOx@[Cu(tz)]/laser (v). Reproduced with permission [94]. Copyright 2022, Wiley. (**F**) CuSACO single-atom nanozyme enables bioorthogonal tumor targeting, catalytic ROS amplification, copper release, and cuproptosis-associated therapy. (a) Synthesis scheme of CuSA and DBCO-modified CuSACO. (b) Fluorescence images of ROS generation. (c) In vivo bioluminescence images of tumor inhibition in glioma-bearing mice. Reproduced with permission [97]. Copyright 2024, Wiley. (**G**) CAPSH sustains copper stress by combining Cu_9_S_8_-mediated copper release with siATP7A-mediated copper-efflux blockade. (a) Construction scheme of CAPSH. (b, c) Tumor photographs and tumor growth curves in subcutaneous and in situ breast models. Reproduced from Ref. [118] under a Creative Commons license. **Note:** Cu, copper; MACuS, metabolism-aiming Cu_2-x_S nanoagents; GLUT1, glucose transporter 1; PAI, photoacoustic imaging; NIR, near-infrared; ICD, immunogenic cell death; TEM, transmission electron microscopy; ROS, reactive oxygen species; Au@MSN-Cu/PEG/DSF, copper-doped mesoporous silica-coated gold nanorods modified with polyethylene glycol and loaded with disulfiram; PEG, polyethylene glycol; DSF, disulfiram; CuET, copper diethyldithiocarbamate; ES, elesclomol; CuO, copper oxide; DLAT, dihydrolipoamide S-acetyltransferase; CuO_2_, copper peroxide; H_2_O_2_, hydrogen peroxide; Cu^2+^, cupric copper; UV–vis, ultraviolet–visible; GOx, glucose oxidase; Cu(I), cuprous copper; [Cu(tz)], copper(I)-tetrazolate coordination polymer; ZIF-8, zeolitic imidazolate framework-8; CuSACO, copper single-atom nanozyme modified with DBCO; CuSA, copper single-atom nanozyme; DBCO, dibenzocyclooctyne; CAPSH, Cu_9_S_8_-based siATP7A-loaded nanoplatform; Cu_9_S_8_, copper sulfide; siATP7A, small interfering RNA targeting ATPase copper transporting alpha. (For interpretation of the references to color in this figure legend, the reader is referred to the Web version of this article.)

Copper-source nanomaterials provide the most direct route to intracellular copper stress, but different material classes address distinct biological barriers. Copper sulfide and copper-deficient chalcogenide systems are suited to light-controlled cuproptosis because they combine copper delivery with photothermal conversion. Glucose-biomineralized copper-deficient copper sulfide (Cu_2-x_S) nanoagents exploit glucose transporter 1 (GLUT1)-mediated tumor uptake and enable second near-infrared (NIR-II) window photothermal enhancement of cuproptosis and immune activation. Related platforms use photothermal heating to synchronize local hyperthermia with copper release [83,85] (Fig. 3A and B). Type-I aggregation-induced emission (AIE) photosensitizer-loaded biomimetic systems further show that copper-containing phototherapeutic platforms can integrate cuproptosis with antimetastatic immunity and protection against tumor rechallenge [84].

Copper oxide (CuO), cuprous oxide (Cu_2_O), and copper peroxide (CuO_2_) platforms provide more direct routes to intracellular copper release [[86], [87], [88], [89], [90]]. Elesclomol-loaded CuO nanoplatforms combine a copper reservoir with enhanced mitochondrial copper transport to promote cuproptosis and antitumor immunity [86] (Fig. 3C). CuO can also induce cuproptosis- and ferroptosis-associated mitochondrial injury, whereas Cu_2_O nanocomposites combine enhanced copper influx, restricted copper efflux, glutathione (GSH) consumption, photothermal therapy (PTT), and chemodynamic therapy (CDT) to sustain copper stress in breast cancer [87,88]. CuO_2_ enables a self-supplying cascade by releasing both Cu^2+^ and hydrogen peroxide (H_2_O_2_) under acidic conditions, thereby linking copper delivery to chemodynamic amplification. Lipo-Ele@CuO_2_ further integrates this chemistry with mitochondrial copper shuttling and radiotherapy sensitization [89,90] (Fig. 3D). Copper-based metal–organic frameworks (MOFs) and coordination polymers are attractive for programmable co-delivery because their high copper content, degradability, and tunable architecture allow copper release to be coordinated with apoptosis, disulfidptosis, pyroptosis, redox disruption, or enzyme-driven therapy [[91], [92], [93], [94],^124^] (Fig. 3E).

Hollow metal-based and bimetallic nano-amplifiers extend this design logic through high internal loading capacity, tumor microenvironment-responsive degradation, and cascade amplification of organelle stress [125]. A copper-dithiocarbamate chelate-doped, artemisinin-loaded hollow platform combined Cu^2+^ release, radical generation, GSH depletion, DLAT oligomerization, and mitochondrial dysfunction to induce cuproptosis-associated injury together with ferroptosis and apoptosis [95]. The hollow calcium/copper bimetallic amplifier D@HCC-CuTH used DSF-loaded CaCO_3_ nanoparticles coated with a copper coordination polymer and hyaluronan. Following tumor-cell uptake, DSF and Cu^2+^ formed a dithiocarbamate–copper complex, intensified endoplasmic reticulum (ER) stress, promoted Ca^2+^ redistribution, and amplified mitochondrial Ca^2+^ overload, ROS generation, and DLAT oligomerization, resulting in coordinated cuproptosis, paraptosis, and apoptosis [96]. These platforms demonstrate the potential of hollow architectures to integrate copper release with redox and organelle stress, although their added complexity is justified only when each component provides independently verified mechanistic value.

Single-atom and nanozyme-based copper systems derive their activity primarily from catalytic efficiency rather than high copper loading. Bioorthogonal Cu single-atom nanozymes, Co–N–C copper-delivering nanozymes, and biodegradable self-cascading nanozymes can promote cuproptosis at relatively low copper doses by amplifying oxidative stress, disrupting metabolism, and depleting GSH [[97], [98], [99]] (Fig. 3F). Organic coordination assemblies and bioinspired copper regulators may be preferable when mild synthesis, tunable coordination chemistry, and biological tolerability are priorities. Dynamic copper–polyphenol nanoparticles, atovaquone-coordinated platforms, and copper-homeostasis nanoregulators illustrate how copper delivery can be integrated with immunomodulation, mitochondrial metabolic disruption, mild photothermal activation, or ferroptosis [[100], [101], [102]]. Platform selection should therefore be guided by the dominant therapeutic barrier, including inadequate release control, limited mitochondrial access, insufficient catalytic amplification, or excessive intracellular copper buffering.

#### 3.1.2 Copper-ionophore delivery and homeostasis reprogramming

Whereas copper-source nanomaterials provide the metal payload directly, copper-ionophore nanodelivery addresses the distinct problems of intracellular copper shuttling and mitochondrial access. In these systems, the carrier stabilizes and localizes a molecule that transports copper across membranes and directs it toward cuproptosis-relevant compartments [19,86,[106], [107], [108], [109], [110]]. Elesclomol remains the clearest mechanistic example because its activity is directly linked to mitochondrial copper delivery and FDX1-dependent cuproptosis [11,126]. Nanoformulation therefore does more than improve pharmacokinetic behavior; it converts a pharmacologically fragile ionophore into a spatially controllable copper-shuttling module [29,79].

ROS-responsive elesclomol–copper nanoparticles favor activation in ROS-rich tumors and enhance the response to anti-PD-L1 therapy [19]. Elesclomol-loaded CuO platforms further integrate a copper reservoir with mitochondria-directed ionophore activity to amplify cuproptosis and antitumor immunity [86]. Natural-product-derived ionophores provide an additional strategy. Lu et al. developed celastrol–copper nanoparticles (Cel-Cu NPs), in which celastrol acted as both a copper ionophore and a GSH-scavenging agent. This dual activity increased intracellular copper availability, weakened thiol-based buffering, promoted DLAT aggregation, and generated self-amplified cuproptosis that enhanced immunogenic cell death and sensitized tumors to anti-PD-L1 therapy [111]. Elesclomol–copper nanoparticles have also been used to overcome multidrug resistance, while elesclomol–copper treatment targets mitochondrial metabolism in pancreatic cancer stem cells, supporting ionophore-based delivery in metabolically resistant tumor states [106,109].

DSF and copper diethyldithiocarbamate (CuET) are established copper-dependent antitumor agents, but their free-drug performance is constrained by instability, rapid metabolism, and insufficient tumor retention [22,110,127]. Nanoformulation can protect DSF or CuET, improve tumor accumulation, and enable stimulus-responsive activation. Apoferritin has been used to deliver Cu(II) diethyldithiocarbamate through a GSH-responsive formulation [107], while stabilized DSF–copper nanoparticles have shown activity against drug-resistant prostate cancer [108]. Folate receptor-targeted poly(lactic-co-glycolic acid)–polyethylene glycol nanoparticles have also improved DSF delivery to breast cancer cells [110]. Inhalable DSF–Cu nanoplatforms further demonstrate how local formulation can restrict ionophore activity, amplify cuproptosis, and connect tumor killing with cGAS–STING activation and immune-checkpoint sensitization [79]. Ionophore nanodelivery is therefore best understood as a control strategy that integrates tumor-site activation, intracellular redox gating, and mitochondrial copper transport.

Beyond material class, release kinetics and copper speciation determine whether delivery produces sustained cuproptotic pressure or transient intracellular accumulation. Slow release may permit buffering and efflux pathways to adapt, whereas excessively rapid release may compromise selectivity before tumor localization is achieved [10,17]. An effective formulation should therefore minimize copper leakage during circulation and trigger rapid activation after tumor uptake. Copper speciation is also important because the reduction of ionophore-bound Cu^2+^ to Cu ^+^ may facilitate copper release and pathway engagement within mitochondria [10]. Platforms that deliver Cu^+^ directly or generate it efficiently at the relevant intracellular site may achieve stronger effects at lower total copper doses [31,128]. Cu_2_O nanocomposites illustrate this principle by combining cuprous copper release with photothermal and chemodynamic effects, enhanced copper influx, GSH consumption, and reduced copper efflux [88].

Copper delivery becomes more effective when it is paired with disruption of the endogenous pathways that would otherwise neutralize it. Nanoparticle uptake may circumvent CTR1-dependent entry constraints, ATP7A/ATP7B interference can reduce copper efflux, and GSH-consuming chemistries can weaken thiol-based buffering [44,47,55]. The objective is not to target multiple pathways indiscriminately, but to prevent therapeutic copper from being buffered or exported before sustained mitochondrial exposure is achieved.

Several platforms have adopted this copper-network-reprogramming strategy. Cascade-targeting copper-homeostasis nanoregulators coordinate tumor-selective copper delivery with mild photothermal activation and ferroptosis–cuproptosis induction [102]. Polydopamine-based copper-deposition nanostructures increase intracellular copper retention by inhibiting copper export and thereby enhance cuproptosis-associated immunotherapy [117]. Cu_9_S_8_-based multifunctional platforms similarly limit the ability of breast cancer cells to restore copper homeostasis after nanoparticle uptake [118] (Fig. 3G). Mechanistic evidence further indicates that ATP7A-dependent copper handling supports mutant Kirsten rat sarcoma viral oncogene homolog (KRAS)-driven lung cancer, whereas ATP7A inhibition disrupts copper homeostasis and increases cuproptosis susceptibility [119]. Thus, copper-network reprogramming may distinguish durable mitochondrial copper stress from transient intracellular copper exposure.

Any strategy that increases tumor copper stress must also limit systemic toxicity. The liver is a major concern because it is central to copper metabolism and nanoparticle clearance [2,17]. Particle-size optimization, ligand-directed uptake, prolonged circulation, and near-neutral surface properties remain important [27,129,130], but they are unlikely to provide adequate protection alone. Cuproptosis nanomedicine should instead adopt a dual-control design in which copper remains relatively inert during circulation and becomes rapidly reactive only after tumor localization and intracellular activation [131,132]. Excessive premature reactivity may damage blood or healthy tissues, whereas excessive stabilization may reduce activity after uptake. Therefore, tumor-activated copper exposure represents a more clinically credible endpoint than high systemic copper exposure alone.

### 3.2 Mitochondrial targeting and vulnerability amplification

**Fig. 4:**
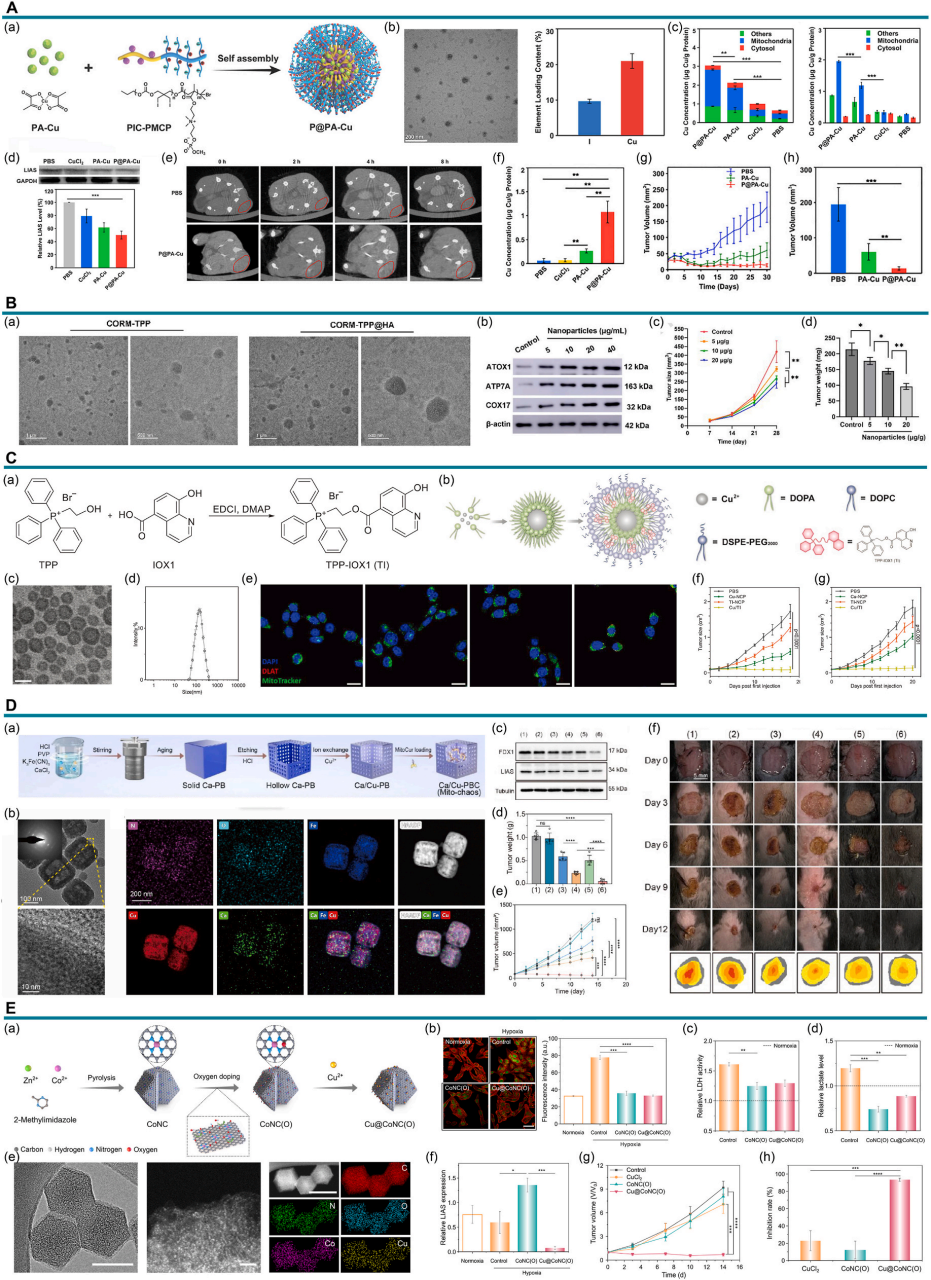
**Mitochondrial targeting and vulnerability amplification in cuproptosis-oriented nanomedicine.** (**A**) P@PA-Cu nanoparticles use pyruvate-Cu as a mitochondria-targeting copper ionophore and an iodine-rich polymer for traceable TNBC therapy. (a) Self-assembly scheme of P@PA-Cu. (b) TEM image and elemental loading analysis. (c) Intracellular and mitochondrial copper distribution. (d) LIAS expression. (e) CT imaging of tumor accumulation. (f) Tumor copper concentration. (g, h) Tumor growth curve and final tumor volume analysis. Reproduced with permission [115]. Copyright 2025, Royal Society of Chemistry. (**B**) CORM-TPP@HA nanoparticles use HA-mediated tumor targeting and TPP-mediated mitochondrial localization to deliver CORM-401 and trigger mitochondria-associated cuproptosis. (a) TEM images of CORM-TPP and CORM-TPP@HA. (b) Western blot analysis of ATOX1, ATP7A and COX17 expression. (c, d) Tumor growth and tumor weight analysis. Reproduced from Ref. [116] under a Creative Commons license. (**C**) Cu/TI nanoscale coordination polymer combines a Cu(II)-containing core with a TPP-IOX1 shell to promote mitochondrial copper delivery and antitumor therapy. (a) Synthesis route of TPP-IOX1. (b) Schematic construction of Cu/TI nanoparticles. (c, d) TEM image and size distribution of Cu/TI. (e) Mitochondrial localization. (f, g) Tumor growth curves in CT26 and 4T1 tumor models. Reproduced from Ref. [113] under a Creative Commons license. (**D**) Mito-chaos amplifies cuproptosis by delivering Cu^2+^ and Ca^2+^ into mitochondria to induce dual-ion perturbation. (a) Preparation scheme of Ca/Cu-PBC-based Mito-chaos. (b) TEM and elemental mapping images. (c) Western blot analysis of FDX1 and LIAS expression. (d, e) Tumor weight and tumor growth curves in postoperative melanoma models. (f) Representative wound images and thermal maps. Reproduced with permission [114]. Copyright 2025, American Chemical Society. (**E**) Cu@CoNC(O) single-atom nanozyme enhances cuproptosis by combining copper delivery with hypoxia relief and metabolic reprogramming. (a) Synthesis and oxygen-doping scheme of Cu@CoNC(O). (b) Hypoxia staining. (c, d) LDH activity and lactate production. (e) TEM, HAADF-STEM, and elemental mapping images of Cu@CoNC(O). (f) LIAS expression. (g, h) Tumor growth curve and tumor inhibition rate. Reproduced with permission [98]. Copyright 2025, American Chemical Society. **Note:** Cu, copper; P@PA-Cu, pyruvate-copper-loaded iodine-rich polymer nanoparticles; TNBC, triple-negative breast cancer; TEM, transmission electron microscopy; LIAS, lipoyl synthase; CT, computed tomography; CORM, carbon monoxide-releasing molecule; TPP^+^, triphenylphosphonium; HA, hyaluronic acid; ATOX1, antioxidant 1 copper chaperone; ATP7A, ATPase copper transporting alpha; COX17, cytochrome *c* oxidase copper chaperone 17; Cu/TI, copper/TPP-IOX1 nanoscale coordination polymer; IOX1, 5-carboxy-8-hydroxyquinoline; Cu^2+^, cupric copper; Ca^2+^, calcium ion; Ca/Cu-PBC, calcium/copper-doped Prussian blue cube; FDX1, ferredoxin 1; Cu@CoNC(O), copper-loaded oxygen-functionalized cobalt–nitrogen–carbon single-atom nanozyme; LDH, lactate dehydrogenase; HAADF-STEM, high-angle annular dark-field scanning transmission electron microscopy. (For interpretation of the references to color in this figure legend, the reader is referred to the Web version of this article.)

Cuproptotic efficacy depends not only on copper delivery, but also on whether copper reaches the mitochondrial compartment in which the pathway is executed. Because lipoylated proteins and Fe–S-dependent machinery are concentrated in mitochondria, whole-cell copper uptake is only an incomplete measure of biological effect. The critical variable is mitochondrial copper exposure in tumor cells that remain metabolically and biochemically permissive for cuproptosis, including retention of a substrate state compatible with lipoylated protein aggregation and Fe–S destabilization [10,98,113,115,[133], [134], [135], [136]]. Mitochondria-centered design requires more than organelle localization. It also requires alignment of copper delivery with the substrate, buffering, and metabolic states that determine whether mitochondrial copper exposure becomes lethal rather than incidental [[113], [114], [115], [116]]. This principle has been reinforced by traceable pyruvate–Cu nanoparticles, in which pyruvate was used as a biocompatible mitochondria-targeting copper ionophore to direct copper stress toward mitochondrial metabolism in triple-negative breast cancer [115] (Fig. 4A). Mitochondria-targeted carbon monoxide-releasing molecule 401 (CORM-401) nanoparticles further support this strategy, as CORM-TPP@HA integrates triphenylphosphonium (TPP^+^)-mediated mitochondrial targeting with hyaluronic acid (HA)-assisted tumor-cell targeting to inhibit A549 cells through cuproptosis [116] (Fig. 4B). This has direct consequences for nanoplatform design, because systems that increase whole-cell copper uptake without improving mitochondrial access may still cause broad oxidative injury while failing to generate efficient cuproptotic execution. By contrast, even modest gains in mitochondrial delivery may substantially alter the relationship between total copper dose and authentic cuproptotic stress [98,113,115,[133], [134], [135]].

This rationale explains why mitochondria-centered design has become a core strategy in cuproptosis-based nanomedicine. Because the key molecular substrates of cuproptosis are localized within mitochondria, cytosolic copper can be buffered by glutathione and metallothioneins or diverted through competing trafficking pathways [1,39,49]. Mitochondria-directed copper delivery offers both spatial precision and improved dose efficiency by limiting intracellular copper sequestration or misrouting before engagement with cuproptosis-relevant substrates. Recent studies of mitochondria-targeted copper platforms suggest that organelle-directed delivery can strengthen cuproptotic efficacy even without a large increase in total intracellular copper [113,115]. Triphenylphosphonium remains the most widely used mitochondrial targeting motif because it provides a practical route to membrane-potential-driven organelle accumulation [133,136,137]. In addition to conventional TPP-based systems, pyruvate–Cu nanoparticles illustrate a metabolism-compatible targeting strategy in which pyruvate functions as both a mitochondrial trafficking element and a copper-shuttling module [115] (Fig. 4A). CORM-TPP@HA nanoparticles also highlight the value of combining mitochondrial targeting with tumor-cell targeting, because TPP promotes mitochondrial localization whereas hyaluronic acid improves tumor-cell uptake through CD44-related targeting [116] (Fig. 4B). Peptide-based approaches, including mitochondria-penetrating peptides and Szeto–Schiller-derived motifs, offer complementary advantages and may be particularly useful in formulations where biological adaptability is preferred over simple lipophilic cation chemistry [134,138,139]. Dual-targeting strategies are also attractive because they can separate tissue-level and organelle-level selectivity, thereby improving both tumor uptake and post-endosomal mitochondrial routing [25,140]. Mitochondria-targeted multifunctional nanoparticles that combine cuproptosis with PD-1 downregulation further demonstrate how organelle-level copper delivery can be integrated with immune modulation, converting mitochondrial copper injury into a broader immunotherapeutic response [113] (Fig. 4C). A mitochondria-targeted dual-ion perturbator similarly shows that subcellular ion disruption can amplify cuproptosis, enhance melanoma immunotherapy and support postoperative wound healing, underscoring the therapeutic value of precisely amplifying mitochondrial dysfunction rather than merely increasing bulk copper exposure [114] (Fig. 4D). However, the presence of a targeting ligand or fluorescence overlap with mitochondria is not sufficient to show that biologically meaningful mitochondrial copper exposure has increased. More convincing studies should combine organelle-specific copper quantification with mechanistic readouts such as DLAT aggregation, Fe–S loss, and mitochondrial proteotoxic stress [113,[133], [134], [135]].

Even efficient mitochondrial access may remain insufficient if reductive buffering is not weakened at the same time. Glutathione is especially important because it can neutralize copper before mitochondrial stress becomes sustained while also supporting broader redox tolerance in stressed tumor cells [40,53,54,141]. MnO_2_-based modules, glutathione-reactive copper systems, and inhibition of glutathione synthesis can all lower the threshold for productive copper stress [[142], [143], [144]]. The timing of glutathione depletion is also important, because copper released before meaningful weakening of the glutathione barrier may remain largely non-productive. Sequential or tightly coupled designs may be preferable when glutathione depletion must precede copper release. In many settings, glutathione depletion is not merely an auxiliary enhancer of cuproptosis, but part of what allows mitochondrial copper exposure to become effective in the first place [113,118,131]. This concept is particularly relevant for mitochondria-targeted platforms that combine copper delivery with immune or ionic modulation, because mitochondrial copper accumulation must occur in a redox environment permissive for lipoylated-protein aggregation and Fe–S protein destabilization [113,114].

Beyond buffering, susceptibility to cuproptosis is also strongly shaped by metabolic state. Oxidative tumor cells are generally more vulnerable, whereas glycolytic tumors and hypoxic subclones can evade copper-induced mitochondrial collapse [10,[145], [146], [147]]. Metabolic priming is most relevant when glycolytic adaptation or hypoxia limits mitochondrial pathway competence. Glycolysis inhibition, reinforcement of pyruvate entry into mitochondria, suppression of HIF-1α-driven adaptation, and glucose oxidase (GOx)-mediated glucose depletion can all shift tumors toward a more cuproptosis-permissive state [145,[147], [148], [149]]. Mitochondria-targeting pyruvate-Cu nanoparticles and copper-delivering single-atom nanozymes that enhance mitochondrial metabolism both suggest that susceptibility can be actively expanded rather than passively selected [98,115] (Fig. 4A–E). The pyruvate–Cu strategy is especially relevant here because it directly links metabolic substrate handling with mitochondrial copper delivery, suggesting that cuproptosis sensitivity can be increased by using metabolism-compatible copper ionophores rather than relying solely on synthetic lipophilic cations [115]. At the same time, some tumors may remain resistant despite successful copper delivery if the susceptible substrate state has already been compromised. Low FDX1 activity, reduced lipoylation, and adaptive remodeling of mitochondrial metabolism can all limit execution despite adequate copper exposure [10,16,62,150]. Future platforms may therefore need to move beyond delivery and sensitization to address substrate competence itself. Although RNA-based restoration of cuproptotic regulators remains at an early stage, continuing advances in lipid nanoparticle-based nucleic acid delivery suggest that this strategy may become increasingly feasible. At the same time, ROS-amplifying copper systems may intensify mitochondrial proteotoxicity and accelerate the collapse of lipoylated and Fe–S-dependent networks once copper reaches the organelle [90,151]. Taken together, effective cuproptotic execution depends not only on copper availability, but also on mitochondrial vulnerability at the levels of buffering, metabolic state, and substrate competence. Designs that assume mitochondrial substrate competence remains intact may underperform in resistant tumors even when copper delivery appears successful.

### 3.3 Stimuli-responsive design and platform engineering

Even when copper delivery is biologically coherent and mitochondrial vulnerability has been amplified, platform performance still depends on whether these effects can be maintained under in vivo conditions. Stimuli-responsive design and broader platform engineering are therefore important at this stage. The central objective is to localize copper activation to tumors without compromising circulation stability, analytical characterization or scalable manufacturing [24,25,31,131,[152], [153], [154], [155], [156]].

Endogenous tumor microenvironment-responsive systems remain attractive because they offer the possibility of coupling tumor entry to intracellular activation without requiring external intervention. Acidic pH, endolysosomal maturation, high intracellular glutathione, elevated peroxide tone, and tumor-associated enzymes can all be used to trigger copper release or carrier disassembly [25,53,90,142,151]. However, the presence of these triggers alone does not guarantee tumor selectivity. Their value depends on whether the activation threshold is tuned well enough to bias reactive copper exposure toward tumors after systemic administration. This is especially important in cuproptosis nanomedicine because premature copper activation may generate redox injury without improving mitochondrial delivery. Hu et al. assembled a chemically defined Cu(I) complex with a ROS-sensitive polymer. Oxidative polymer cleavage triggered Cu(I) release in ROS-rich tumor cells, increasing mitochondrial copper accumulation, DLAT oligomerization and cuproptosis. The same platform promoted dendritic-cell maturation and CD8^+^ T-cell infiltration, linking responsive copper activation to antitumor immunity [112]. Endogenous responsiveness is therefore useful only when it creates a meaningful selectivity window in vivo.

Exogenous activation can provide stronger temporal and spatial control when endogenous selectivity is insufficient. Near-infrared-triggered copper sulfide systems remain particularly attractive because they combine local heating, accelerated copper release, and mitochondrial stress within a single intervention [157,158]. Ultrasound- and radiotherapy-linked activation broaden the available trigger space and may be particularly relevant for deep or clinically accessible lesions [159,160]. Magnetic guidance is also conceptually interesting, although its practical depth limitations remain substantial [25]. The main value of these external triggers lies in narrowing the gap between tumor copper activation and systemic copper exposure. In this setting, the most useful exogenous triggers are likely to be those that synchronize copper release with local mitochondrial vulnerability rather than simply impose an additional source of generalized injury [31,131]. Once activation has been spatially controlled, cascade amplification offers a further level of engineering. Copper can be incorporated into self-reinforcing biochemical sequences involving glucose depletion, peroxide generation, glutathione exhaustion, Cu^+^ formation, ROS amplification, and mitochondrial proteotoxicity [148,149]. This creates the possibility that limited initial activation may be translated into broader biochemical collapse. However, each additional step also introduces kinetic bottlenecks, stoichiometric constraints, and translational cost. A cascade is therefore useful only when each step resolves a genuine biological barrier and intermediate states are measured rather than inferred solely from endpoint cytotoxicity [98].

Beyond activation logic, in vivo performance also depends on how effectively the platform manages circulation behavior, tissue access, and premature copper reactivity. Biomimetic formulations remain of interest because they can improve circulation, reduce clearance, and may also enhance lesion localization in selected contexts. Erythrocyte membranes, cancer cell membranes, leukocyte-derived coatings, and exosome-inspired systems each provide a different biological interface [[161], [162], [163], [164], [165], [166], [167]]. In cuproptosis nanomedicine, their main value is to shield reactive copper during circulation while preserving tumor access and intracellular release. However, biomimetic design is most persuasive when it offers a clear functional advantage rather than added conceptual complexity. These systems can increase variability and analytical demands. They are best suited to situations in which they solve a well-defined in vivo delivery problem that simpler surface-engineering approaches cannot address [166,167]. The same principle applies to surface engineering more broadly. PEGylation can improve circulation and reduce rapid immune recognition, but the PEG dilemma remains relevant because the same protective corona may also limit tumor uptake and intracellular delivery [168,169]. Cleavable PEG, stimulus-removable stealth layers, and related surface-switching strategies therefore remain highly relevant [169,170]. In copper-based systems, protein corona formation may influence biodistribution, cellular uptake, thiol accessibility, and even premature redox activity at the nanoparticle surface [[171], [172], [173], [174]]. Surface engineering in cuproptosis nanomedicine should therefore be judged not only by circulation half-life, but also by whether it suppresses unwanted copper chemistry before tumor uptake and while still permitting efficient intracellular activation.

Taken together, these considerations lead to a broader translational question: how much mechanistic sophistication can be incorporated without undermining development discipline? Many cuproptosis nanoplatforms now integrate copper sources, ionophores, targeting ligands, redox-active modules, metabolic modulators, responsive shells, and immune components within a single construct [24,25,152]. Such systems can be compelling in proof-of-concept studies, but they also increase formulation complexity, batch variability, analytical burden, and regulatory uncertainty. Experience in successful nanomedicine development suggests that therapeutic benefit more often comes from solving a limited number of delivery problems with clear pharmacological value than from increasing platform complexity [131,153,154,156,175,176]. For translation, multifunctionality should be limited to components with a defined and measurable contribution to delivery, pathway engagement or safety. Each added element should address a defined biological barrier and make a clear contribution to copper bioavailability, mitochondrial access, buffering depletion, susceptibility expansion, or in vivo selectivity. The aim is not to make cuproptosis platforms progressively more elaborate, but to develop the simplest design that can reproducibly translate tumor copper dependence into tumor-selective mitochondrial injury [131,156,174,[177], [178], [179]]. This principle is likely to be central if cuproptosis nanomedicine is to move from elegant preclinical chemistry toward clinically meaningful benefit.

### 3.4 Copper depletion and copper-disequilibrium therapy

Copper-depleting nanomedicine should be distinguished from copper-supplying cuproptosis platforms. Canonical cuproptosis is generally driven by copper accumulation, engagement of lipoylated mitochondrial proteins, aggregation of DLAT or related TCA-cycle proteins, destabilization of Fe–S proteins, and mitochondrial proteotoxic stress [10,12,16]. In contrast, copper-depleting strategies reduce bioavailable copper pools or disrupt copper-dependent tumor biology [180]. These approaches create copper disequilibrium through depletion rather than overload and should be discussed separately from canonical cuproptosis. This distinction is important because copper depletion may suppress tumor growth through several mechanisms, including inhibition of copper-dependent mitochondrial respiration, angiogenesis, extracellular matrix remodeling, metastatic niche formation, and copper-dependent signaling. These effects may be therapeutically valuable, but they should not be described as canonical cuproptosis unless pathway-specific evidence, including FDX1 dependence, lipoylated DLAT aggregation, Fe–S protein loss, and appropriate rescue experiments, is provided.

The rationale for copper depletion is supported by the dependence of many tumors on copper-regulated bioenergetic and microenvironmental processes. Mitochondrial copper is required for cytochrome *c* oxidase activity and oxidative phosphorylation, making oxidative tumor states potentially vulnerable to selective copper deprivation. Cui et al. developed a mitochondria-targeted copper-depleting nanoparticle for triple-negative breast cancer. This system decreased oxygen consumption and oxidative phosphorylation, shifted metabolism toward glycolysis, reduced ATP production, disrupted mitochondrial membrane potential, increased oxidative stress, and suppressed tumor growth in mouse models [121]. This study is mechanistically distinct from copper-overload cuproptosis, because tumor suppression was mainly attributed to mitochondrial copper deprivation, energy deficiency, and apoptosis. Nevertheless, it demonstrates that selective disruption of mitochondrial copper availability can be a powerful therapeutic strategy for tumors with high mitochondrial dependence.

Recent work has extended this concept to copper-consuming nanomedicine. Zhou et al. reported ZnS nanoparticles as a smart copper-depleting nanodrug that consumes copper ions through a cation exchange reaction driven by the large difference in solubility product constants between ZnS and CuS [120]. The resulting copper depletion was reported to induce tumor cell death both in vitro and in vivo and was described as copper depletion-induced tumor cuproptosis. This study broadens the therapeutic landscape by showing that copper disequilibrium can be engineered not only by copper delivery, but also by local copper consumption. However, because this mechanism proceeds through copper removal rather than copper overload, it should be interpreted carefully. For this class of materials, future studies should define whether the observed death program fully converges on canonical FDX1–DLAT cuproptosis or instead reflects a related copper-disequilibrium state involving mitochondrial dysfunction, impaired copper enzymes, and secondary proteotoxic or metabolic stress.

Copper-depletion therapy also has translational relevance beyond nanomedicine. Tetrathiomolybdate, a systemic copper-depleting agent, has been evaluated in patients with high-risk breast cancer and in preclinical metastasis models. In a phase II study, tetrathiomolybdate was used to maintain low ceruloplasmin levels and was associated with modulation of copper-dependent tumor microenvironmental components, including reduced endothelial progenitor cells and lysyl oxidase-like 2 (LOXL2), with acceptable tolerability [122]. Related studies further showed that tetrathiomolybdate-associated copper depletion can affect collagen remodeling and metastatic niche formation, consistent with the copper dependence of LOX/LOXL enzymes and extracellular matrix organization [123]. These findings suggest that copper depletion may be especially relevant for metastasis prevention, tumor microenvironment modulation, and combination therapy, even when it does not directly induce canonical cuproptosis.

Overall, copper-depleting nanomedicine should be framed as an inverse copper-disequilibrium strategy that complements, but should not be conflated with, copper-supplying cuproptosis nanomedicine. Its potential advantages include reduced risk of copper-overload toxicity, selective disruption of copper-dependent tumor metabolism, and modulation of metastatic or immune-permissive microenvironments. Its major challenges include maintaining tumor selectivity, avoiding systemic copper deficiency, defining organ-specific copper depletion, and establishing whether cell death is truly cuproptotic or instead mediated by apoptosis, metabolic collapse, anti-angiogenic effects, or broader copper starvation. Future studies should therefore combine copper quantification, mitochondrial copper imaging, ceruloplasmin or copper-burden monitoring, respiratory-chain analysis, DLAT aggregation, Fe–S protein assessment, and genetic or pharmacological rescue experiments to distinguish canonical cuproptosis from copper-depletion-associated tumor suppression.

## 4 Combination therapeutic strategies

**Fig. 5:**
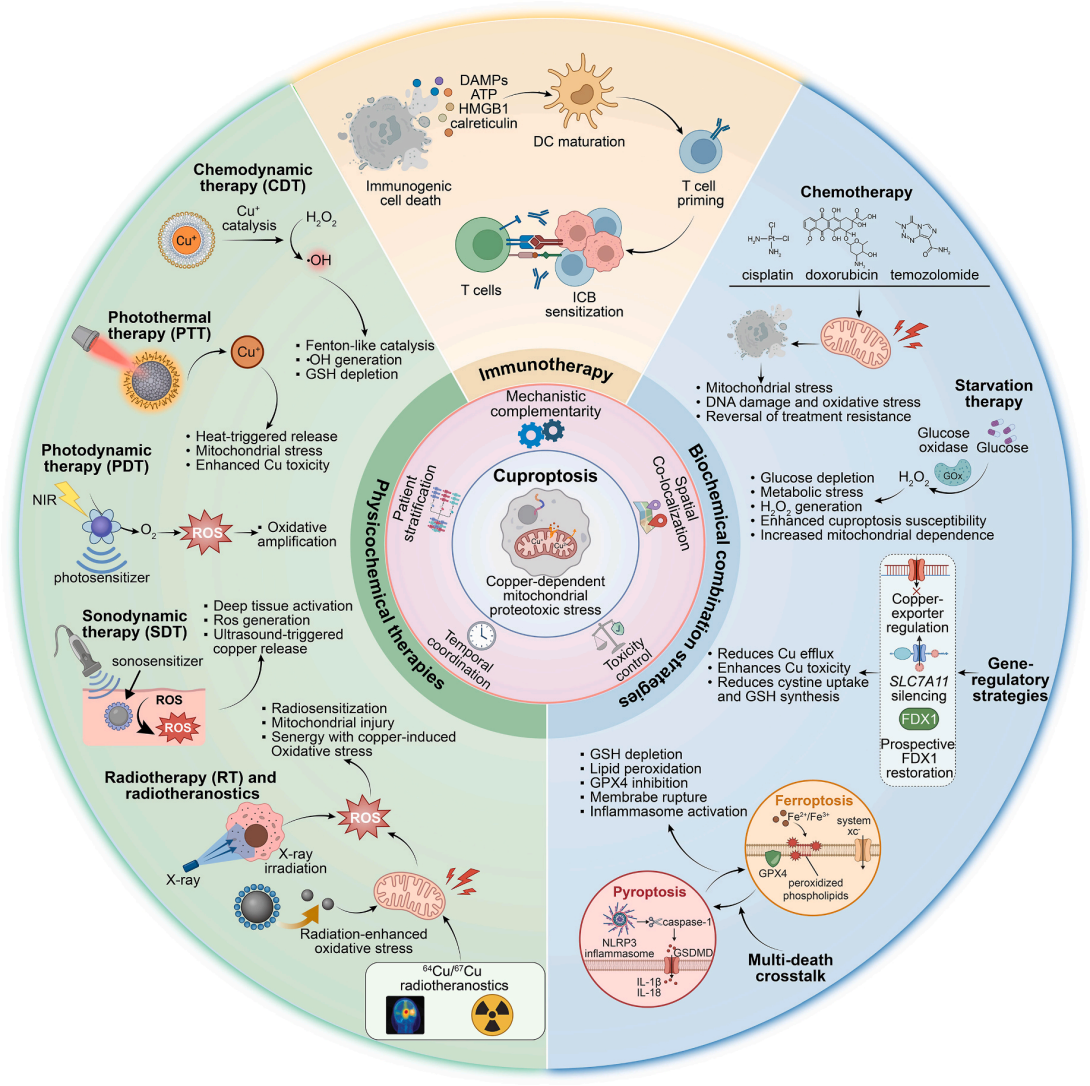
**Cuproptosis-centered combination therapeutic strategies.** The wheel-shaped schematic summarizes three major combination frameworks for cuproptosis nanomedicine, guided by mechanistic complementarity, spatial co-localization, temporal coordination, toxicity control, and patient stratification. Physicochemical therapies include chemodynamic, photothermal, photodynamic, sonodynamic, and radiation-based approaches, together with copper radiotheranostics. These strategies enhance cuproptosis through Cu^+^-catalyzed Fenton-like reactions, reactive oxygen species generation, heat-triggered copper release, mitochondrial injury, and radiation-enhanced oxidative stress. Immunotherapy-oriented combinations may exploit cuproptosis-associated immunogenic signaling, DAMP release, dendritic-cell maturation, T-cell priming, and sensitization to immune checkpoint blockade. Biochemical combination strategies include chemotherapy, starvation therapy, gene-regulatory strategies, and crosstalk with ferroptosis and pyroptosis. Chemotherapy reinforces mitochondrial and oxidative stress; starvation therapy promotes glucose depletion and H_2_O_2_ generation and may increase reliance on mitochondrial metabolism; gene-regulatory strategies may suppress copper efflux, weaken antioxidant buffering, or reduce immune escape, whereas FDX1 restoration remains a prospective approach for sensitizing FDX1-low tumors to cuproptosis. Crosstalk with ferroptosis and pyroptosis involves glutathione depletion, lipid peroxidation, GPX4 inhibition, membrane rupture, and inflammasome activation. **Note:** ATP, adenosine triphosphate; ATP7A/ATP7B, ATPase copper transporting alpha/beta; CDT, chemodynamic therapy; DAMPs, damage-associated molecular patterns; DC, dendritic cell; FDX1, ferredoxin 1; GPX4, glutathione peroxidase 4; GSH, glutathione; GSDMD, gasdermin D; H_2_O_2_, hydrogen peroxide; HMGB1, high mobility group box 1; ICB, immune checkpoint blockade; NLRP3, NLR family pyrin domain containing 3; PDT, photodynamic therapy; PTT, photothermal therapy; ROS, reactive oxygen species; RT, radiotherapy; SDT, sonodynamic therapy; SLC7A11, solute carrier family 7 member 11; ^64^Cu-PET, copper-64 positron emission tomography.

Cuproptosis-based nanomedicine may require combination strategies because metabolic heterogeneity, adaptive restoration of copper homeostasis, and immunosuppressive tumor microenvironments can limit monotherapy [10,16,181]. Rational combinations should therefore converge on copper handling, mitochondrial vulnerability, redox imbalance, tumor metabolism, or antitumor immunity rather than merely add unrelated therapeutic functions (Fig. 5). Representative nanoplatform-enabled combinations and their mechanistic rationale are summarized in Table 2. Future studies should emphasize pathway-specific validation, including FDX1 dependence, aggregation of lipoylated proteins, Fe–S protein loss, and appropriate rescue experiments, to distinguish canonical cuproptosis from nonspecific multimodal cytotoxicity.

**Table 2:** Rational combination strategies based on cuproptosis nanomedicine.

Combination strategy	Representative platforms	Mechanistic basis for synergy	Main therapeutic outputs	Potentially suitable tumor contexts	Key limitations	Ref.
Cuproptosis + chemodynamic therapy	CuO_2_, CuS, Cu-MOF, Cu single-atom nanozyme, Cu(I) coordination polymer, GOx/Cu systems, lactate oxidase–Cu systems	Cu^+^ catalyzes Fenton-like reactions; Cu^2+^ consumes GSH and generates Cu^+^; ROS damages mitochondria, Fe–S proteins and lipoylated proteins; GOx or LOx can provide H_2_O_2_ and metabolic stress	Enhanced ROS burst, GSH depletion, mitochondrial dysfunction and copper-triggered proteotoxic stress	GSH-rich, H_2_O_2_-rich, lactate-rich or redox-vulnerable tumors	CDT and cuproptosis readouts may overlap; pathway-specific rescue experiments are required to distinguish canonical cuproptosis from ROS-dominant cytotoxicity	[94,182,183]
Cuproptosis + photothermal therapy	CuS, Cu_2-x_S, and other NIR-responsive copper chalcogenides	Photothermal heating accelerates copper release, damages mitochondria, increases Fenton-like reaction kinetics and facilitates local immune activation	Spatially controlled tumor ablation, amplified copper stress, improved immunogenicity	Superficial or endoscopically accessible tumors, breast cancer, melanoma and accessible solid tumors	Limited penetration of NIR light; thermal ablation may obscure the cuproptosis-specific contribution	[83]
Cuproptosis + photodynamic therapy	Cu_2_O@SiO_2-_Ce6 and related photosensitizer–copper nanoplatforms	PDT-derived ROS increase mitochondrial injury and may lower the cuproptosis threshold, while copper stress may provide complementary activity in mitochondria-competent tumor regions	Oxidative amplification, mitochondrial injury and immune activation	Oxygenated tumor regions and tumors accessible to light	PDT is oxygen-dependent; hypoxic tumor cores may respond poorly; ROS-mediated injury must be distinguished from copper-dependent proteotoxic death	[184]
Cuproptosis + sonodynamic therapy	CuO_2_, Cu-MOF, TiO_2_/Cu, hematoporphyrin/Cu systems, copper-nitride nanosonosensitizers and ultrasound-triggered polymeric nanoparticles	Ultrasound activates sonosensitizers, promotes ROS generation and can trigger copper release in deep tissues	Deep-tissue ROS generation plus copper-mediated proteotoxic stress	Deep-seated tumors, visceral tumors and potentially brain tumors	Sonosensitizer efficiency, ultrasound parameters, oxygen dependence and safety need standardization	[[185], [186], [187], [188]]
Cuproptosis + radiotherapy	Copper nitroimidazole, Cu-based radiosensitizing nanoparticles, Cu-MOF/RT systems, Lipo-Ele@CuO_2_ and X-ray-responsive copper systems	Copper-based nanoplatforms may enhance radiotherapy by amplifying ROS and DNA damage, while RT-induced CTR1 upregulation and mitochondrial GSH depletion further increase mitochondrial copper stress	Radiosensitization, oxidative injury, mitochondrial dysfunction and possible immune priming	Radioresistant tumors and selected hypoxic tumors treated with hypoxia-modulating or hypoxia-responsive platforms	Need to separate radiosensitization from true cuproptosis; normal tissue radiation toxicity must be controlled	[89,[189], [190], [191]]
Cuproptosis + immunotherapy	Cuproptosis-inducing nanomedicine combined with anti-PD-1/PD-L1 therapy; Cu(I)-complex nanoparticles; elesclomol–copper nanocarriers; and STING-oriented approaches	Cuproptosis may release DAMPs, promote ICD, stimulate dendritic-cell maturation and remodel immunosuppressive TME; checkpoint blockade prevents adaptive immune escape	Enhanced CD8^+^ T-cell infiltration, macrophage repolarization and improved checkpoint blockade response	“Cold” tumors, immune-excluded tumors, colorectal cancer, renal cancer, melanoma, bladder cancer and immune-resistant breast cancer	ICD evidence remains context-dependent; immune biomarkers and systemic safety must be carefully defined	[19,79,86,112,185,192,193]
Cuproptosis + ferroptosis	Cu/Fe bimetallic nanoparticles, Cu/Mo nanodots, DSF/Cu plus xCT or GPX4 inhibition, Cu plus sorafenib/RSL3 systems, Ce6@Cu nanoplatforms	Shared dependence on GSH depletion, mitochondrial damage and metal-catalyzed oxidative stress; copper and iron redox cycling can jointly promote lipid peroxidation and mitochondrial proteotoxicity	Dual metal-dependent cell death, lipid peroxidation and mitochondrial collapse	Drug-resistant tumors with high GSH or ferroptosis vulnerability	Death pathways may be difficult to distinguish; excessive systemic oxidative toxicity is possible	[187,193]
Cuproptosis + starvation therapy	GOx/Cu nanoparticles, glucose-depleting copper platforms, CuS@GOx@ES and copper-based nanotubes combining 2-DG, elesclomol or copper ionophores	GOx consumes glucose and generates H_2_O_2_, reducing glycolytic flux and potentially increasing reliance on mitochondrial metabolism in metabolically flexible cells	Starvation, CDT amplification and cuproptosis sensitization	Glycolytic or glucose-dependent tumors with adequate nanoparticle delivery and oxygenation	Hypoxia and nutrient heterogeneity may limit efficacy; enzyme stability and GOx immunogenicity require attention	[148,149,194,195]
Cuproptosis + metabolic reprogramming	Copper plus 2-DG, DCA, HIF-1α inhibitors, lactate-depleting systems, p53-reactivating copper platforms, and LOx/Cu biomimetic regulators	Glycolysis blockade, lactate depletion, or mitochondrial metabolic rewiring may increase reliance on OXPHOS and the availability of lipoylated mitochondrial substrates	May sensitize glycolysis-dominant, cuproptosis-resistant cells	Tumors with metabolic plasticity, stem-like features or therapy-resistant populations	Metabolic adaptation may still occur; systemic metabolic toxicity requires monitoring	[38,147,183,196]
Cuproptosis + chemotherapy	Cu nanocarriers co-loaded with doxorubicin or cisplatin/DDP, and GSH-resistant CuET nanomedicine for chemotherapy-resistant tumors. Other chemotherapeutics, including oxaliplatin and paclitaxel, remain potential but less directly validated extensions	Copper stress weakens mitochondrial and redox defenses; chemotherapy increases DNA damage, apoptosis or ROS burden; CuO_2_ can remodel the TME by consuming GSH, relieving hypoxia/acidosis and blocking cisplatin efflux; GSH-resistant CuET bypasses high-GSH-mediated platinum resistance and induces cuproptosis	Overcomes chemoresistance, enhances tumor killing, integrates cuproptosis with apoptosis, CDT or platinum sensitization, and may enable dose reduction	Platinum-resistant NSCLC, cisplatin-resistant tumors, apoptosis-resistant lung cancer, high-GSH tumors and multidrug-resistant tumors	Evidence is strongest for cisplatin/DDP and CuET, moderate for DOX, and currently limited for oxaliplatin and paclitaxel. Pharmacokinetic matching, dose sequencing and additive toxicity require careful optimization	[[197], [198], [199], [200], [201]]
Cuproptosis + gene-regulatory strategies	Copper delivery combined with gene regulation, including PD-L1 siRNA-loaded CuS–PEI–silk fibroin nanoparticles, ATP7A-targeting and broader copper-exporter-regulatory strategies, SLC7A11/xCT silencing for GSH depletion, and prospective FDX1 restoration strategies	Gene regulation may suppress copper efflux, GSH buffering, or immune escape. PD-L1 and SLC7A11/xCT silencing target immune evasion and cystine/GSH metabolism, whereas FDX1 restoration remains prospective in FDX1-low tumors	Mechanism-guided sensitization, reduced copper dose requirement, enhanced immune response and potential reversal of cuproptosis resistance	FDX1-low, copper-efflux-high, GSH-high, and immune-resistant tumors	Evidence is strongest for PD-L1 siRNA nanoplatforms. SLC7A11/xCT silencing is better established in ferroptosis, while FDX1 restoration with copper delivery remains prospective	[[117], [118], [119],^202^]

Note: Cu, copper; CuO_2_, copper peroxide; CuS, copper sulfide; Cu-MOF, copper-based metal–organic framework; Cu(I), cuprous copper; GOx, glucose oxidase; LOx, lactate oxidase; H_2_O_2_, hydrogen peroxide; GSH, glutathione; ROS, reactive oxygen species; Fe–S, iron–sulfur; CDT, chemodynamic therapy; NIR, near-infrared; Cu_2-x_S, copper-deficient copper sulfide; PDT, photodynamic therapy; Cu_2_O, cuprous oxide; Ce6, chlorin e6; SDT, sonodynamic therapy; TiO_2_, titanium dioxide; RT, radiotherapy; CTR1, copper transporter 1; TME, tumor microenvironment; PD-1, programmed cell death protein 1; PD-L1, programmed death-ligand 1; STING, stimulator of interferon genes; DAMPs, damage-associated molecular patterns; ICD, immunogenic cell death; CD8^+^ T cells, cluster of differentiation 8-positive T cells; Cu/Fe, copper/iron; Cu/Mo, copper/molybdenum; DSF, disulfiram; xCT, cystine/glutamate antiporter; GPX4, glutathione peroxidase 4; RSL3, Ras-selective lethal 3; 2-DG, 2-deoxy-D-glucose; OXPHOS, oxidative phosphorylation; DCA, dichloroacetate; HIF-1α, hypoxia-inducible factor 1-alpha; p53, tumor protein p53; DDP, cisplatin; CuET, copper diethyldithiocarbamate; DOX, doxorubicin; DNA, deoxyribonucleic acid; NSCLC, non-small cell lung cancer; FDX1, ferredoxin 1; ATP7A, ATPase copper transporting alpha; SLC7A11, solute carrier family 7 member 11; PEI, polyethyleneimine; siRNA, small interfering RNA.

### 4.1 Cuproptosis and physicochemical therapies

Copper-based nanomaterials are particularly attractive for combination therapy because a single material can often provide both the trigger for cuproptosis and an additional physicochemical therapeutic function. Depending on composition, copper-based materials can support catalytic redox therapy, photothermal conversion or radiation-responsive oxidative amplification. Co-delivery can improve spatial and temporal coordination and avoid the pharmacokinetic mismatch that often limits separately administered combination components. More importantly, these platforms can synchronize copper activation with local biochemical or physical vulnerability, which is especially valuable for a threshold-dependent process such as cuproptosis.

#### 4.1.1 Cuproptosis combined with chemodynamic therapy

Among physicochemical combination strategies, CDT is one of the most closely linked to cuproptosis because both depend on Cu^+^ generation, GSH consumption and mitochondrial redox stress [94,182,183,203,204]. CDT relies on Cu^+^-catalyzed Fenton-like reactions, whereas cuproptosis requires mitochondrial copper engagement with lipoylated proteins. The two processes can intersect through copper redox cycling and glutathione depletion [53,90,143] (Fig. 3D). Reduction of Cu^2+^ to Cu ^+^ can simultaneously support cuproptotic pathway engagement and Fenton-like hydroxyl-radical generation, while glutathione buffering shapes both copper availability and CDT-associated oxidative damage. These shared intermediates and defense mechanisms mean that CDT and cuproptosis function not simply as parallel modalities within the same nanoplatform, but as biochemically coupled processes.

CDT is often limited by insufficient endogenous H_2_O_2_ in tumors [143]. Self-supplying designs are particularly attractive in this setting because co-encapsulation of glucose oxidase with copper-based platforms can couple glucose consumption with H_2_O_2_ generation while simultaneously imposing metabolic stress on glycolysis-dependent cells [148,149,195]. In selected tumor models, glucose depletion may increase dependence on alternative oxidative substrates and enhance sensitivity to mitochondrial stress. However, this metabolic shift should be experimentally validated rather than assumed. Calcium peroxide systems offer an alternative local source of H_2_O_2_ and may further increase mitochondrial stress through Ca^2+^ release [205]. Under these conditions, CDT is likely to be most effective when ROS generation is accompanied by metabolic and redox changes that favor cuproptosis.

Available evidence indicates that CDT–cuproptosis systems operate as mutually reinforcing biochemical circuits rather than as two parallel cytotoxic modalities. Enzyme-engineered Cu(I) coordination polymer nanoplatforms use glucose oxidase-mediated glucose oxidation to increase intratumoral H_2_O_2_ production and local acidity, thereby creating a tumor microenvironment that favors both Cu-dependent Fenton-like ROS generation and copper-triggered cell death. CuO_2_-MSN@TA-Cu^2+^ follows a related cascade mechanism, in which CuO_2_ supplies endogenous H_2_O_2_ and the TA–Cu^2+^ layer provides an additional copper source. After release, Cu^2+^ is reduced by intracellular GSH to Cu^+^, which simultaneously depletes antioxidant buffering, generates the cuproptosis-active copper species, and accelerates Cu^+^-mediated hydroxyl radical production [94,182] (Fig. 3E). Using a biomineralization strategy, Li et al. constructed pH-responsive celastrol–tannic acid–copper nanohybrids (Cel-TA-Cu NP). Acid-triggered disassembly released celastrol and Cu^2+^, after which GSH-mediated Cu^+^ formation promoted Fenton-like hydroxyl radical generation, while celastrol enhanced apoptosis through Bcl-2 degradation and caspase-3 activation [206]. Although canonical cuproptosis was not directly established, this platform illustrates how copper coordination can integrate tumor-responsive delivery with CDT and apoptosis. Biomimetic lactate oxidase-loaded copper nanoregulators provide another metabolic amplification route by depleting lactate, generating H_2_O_2,_ and inducing a lactate-depletion-mediated ROS storm that cooperates with copper-induced cuproptosis and immune remodeling in clear cell renal cell carcinoma [183]. Together, these systems show that CDT and cuproptosis are connected through a shared copper-redox and metabolic-amplification axis. H_2_O_2_ production fuels Cu-dependent radical generation; GSH consumption converts Cu^2+^ to Cu^+^, thereby weakening antioxidant defense; and the resulting oxidative stress further promotes mitochondrial dysfunction, Fe–S protein instability, and lipoylated protein damage. This reciprocal reinforcement provides a strong mechanistic basis for CDT–cuproptosis synergy, because each reaction step increases the probability that the next will occur rather than simply adding another layer of nonspecific cytotoxicity.

#### 4.1.2 Cuproptosis combined with photothermal therapy

Photothermal therapy is particularly well suited to combination with cuproptosis because copper chalcogenides such as CuS and Cu_2_-xS can serve as both copper reservoirs and photothermal agents [83,157,158,207]. The rationale for this combination lies in the intrinsic properties of copper chalcogenides. Localized hyperthermia can accelerate nanoparticle degradation, increase intracellular copper release, intensify Fenton-like reaction kinetics, and pre-stress mitochondria through heat-induced membrane and respiratory dysfunction [143,157,[208], [209], [210]]. Hyperthermia may also transiently increase vascular permeability and improve local nanoparticle penetration [211].

Photothermal irradiation provides an external activation gate by synchronizing local hyperthermia with nanoparticle degradation, copper release, and tumor-restricted copper amplification. This strategy can confine the most damaging phase of copper stress to the irradiated lesion, thereby improving spatial selectivity while reducing systemic exposure. Guo et al. showed that NIR-responsive copper-based nanocrystals achieved stronger tumor regression when photothermal activation was combined with copper release than when either modality was used alone [207]. A related strategy has been demonstrated using glucose-biomineralized Cu_2-x_S nanoagents, which exploit GLUT1-mediated tumor metabolic uptake to improve intracellular copper delivery. Under NIR-II irradiation, these nanoagents produced direct photothermal ablation, promoted copper-dependent cuproptosis, and intensified ROS-mediated cytotoxicity. The combined photothermal, cuproptotic, and oxidative effects further induced immunogenic cell death, activated systemic antitumor immunity, and suppressed tumor growth, metastasis, and recurrence in breast cancer models [83] (Fig. 3A). These findings suggest that copper chalcogenide nanoplatforms can function not only as photothermal agents, but also as metabolically targeted and light-activated copper reservoirs that integrate local tumor ablation, cuproptosis induction, and immune activation within a single therapeutic framework. At present, however, PTT–cuproptosis combinations remain most suitable for superficial, intraoperative, or endoscopically accessible lesions unless deeper-penetrating energy inputs or image-guided activation strategies are introduced.

#### 4.1.3 Cuproptosis combined with photodynamic therapy

**Fig. 6:**
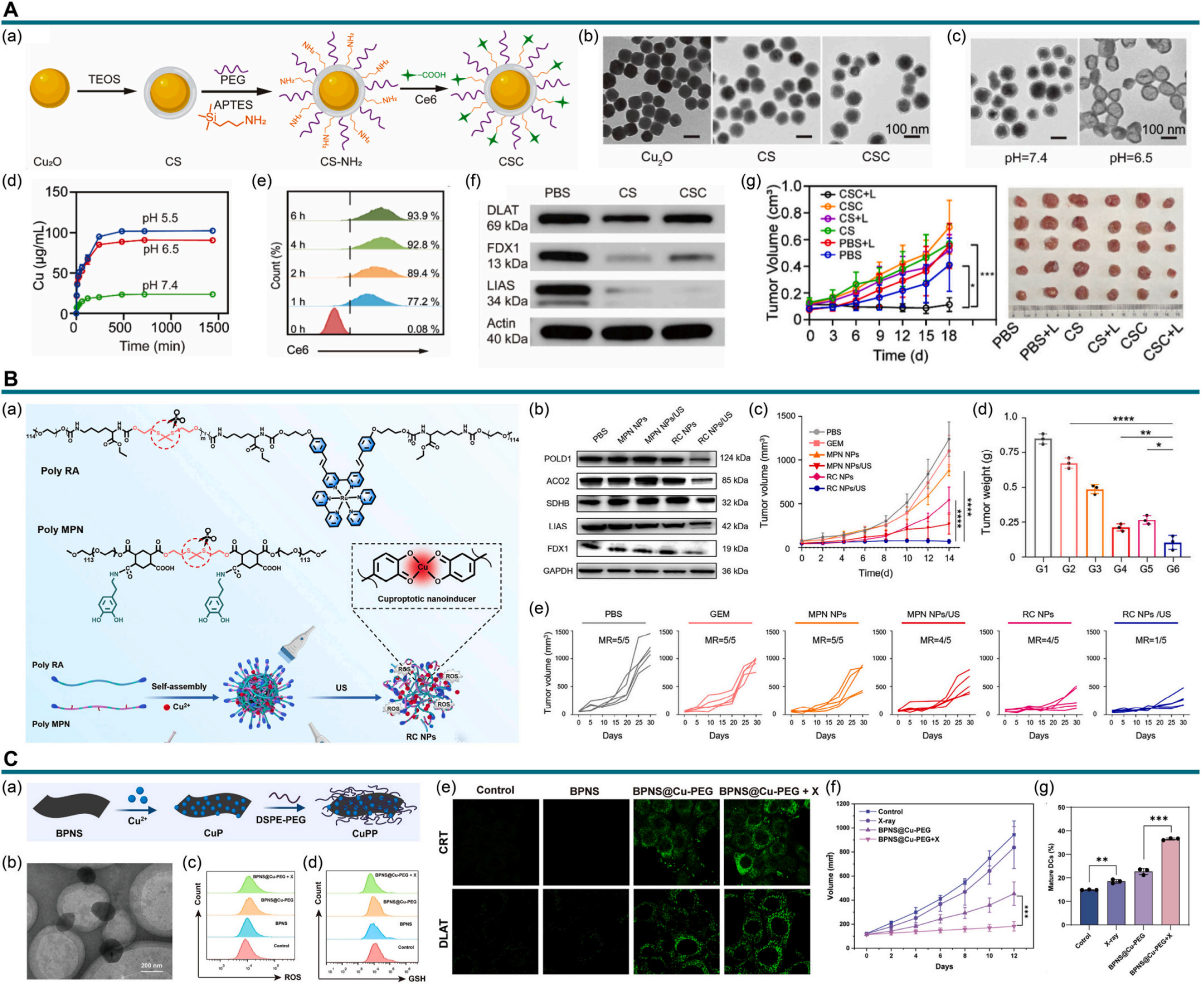
**Physicochemical combination therapies based on cuproptosis.** (**A**) Cu_2_O@SiO_2_-Ce6 integrates acid-responsive copper release with Ce6-mediated photodynamic therapy. (a) Construction scheme. (b) TEM images of Cu_2_O, CS and CSC. (c) pH-responsive degradation. (d) Copper release profile. (e) Cellular uptake of Ce6. (f) DLAT, FDX1 and LIAS expression. (g) Tumor growth curves and tumor photographs. Reproduced with permission [184]. Copyright 2025, Royal Society of Chemistry. (**B**) RC NPs combine ultrasound-triggered sonodynamic therapy with copper release and cuproptosis. (a) Schematic illustration of ROS-responsive RC NPs. (b) Fe–S cluster and cuproptosis-related protein expression. (c–e) Average and individual tumor growth curves and tumor weight analysis. Reproduced with permission [185]. Copyright 2025, Wiley. (**C**) Copper-enriched black phosphorus nanoplatforms sensitize low-dose radiotherapy through cuproptosis and immune activation. (a) Construction scheme of CuP-loaded BPNS. (b) TEM image. (c, d) ROS generation and GSH depletion. (e) CRT and DLAT staining. (f) Tumor growth curves. (g) Mature DC analysis. Reproduced from Ref. [190] under a Creative Commons license. **Note:** Cu, copper; Cu_2_O, cuprous oxide; SiO_2_, silicon dioxide; Ce6, chlorin e6; CS, Cu_2_O@SiO_2_; CSC, Cu_2_O@SiO_2_-Ce6; PDT, photodynamic therapy; TEM, transmission electron microscopy; DLAT, dihydrolipoamide S-acetyltransferase; FDX1, ferredoxin 1; LIAS, lipoyl synthase; RC NPs, ROS-responsive copper nanoparticles; NPs, nanoparticles; SDT, sonodynamic therapy; ROS, reactive oxygen species; Fe–S, iron–sulfur; CuP, copper peroxide; BPNS, black phosphorus nanosheets; RT, radiotherapy; GSH, glutathione; CRT, calreticulin; DC, dendritic cell. (For interpretation of the references to color in this figure legend, the reader is referred to the Web version of this article.)

Upon light activation, photodynamic therapy (PDT) generates singlet oxygen and related ROS, thereby amplifying oxidative injury to mitochondrial membranes, respiratory chain components, and other susceptible macromolecules [[212], [213], [214]]. When combined with cuproptosis, this exogenous ROS burst may increase mitochondrial vulnerability and make copper-triggered proteotoxic collapse more likely. This principle is exemplified by Cu_2_O@SiO_2_-Ce6 nanoparticles, in which a cuprous oxide core is encapsulated within a silicon dioxide shell and covalently linked to the photosensitizer chlorin e6 [184] (Fig. 6A). After tumor accumulation, the acidic tumor microenvironment and lysosomal compartments promote copper ion release from the Cu_2_O core, leading to mitochondrial cuproptosis through aggregation of lipoylated TCA-cycle proteins and depletion of Fe–S cluster proteins. Upon laser irradiation, Ce6 generates cytotoxic ROS, which further disrupts tumor cell membranes and intensifies oxidative injury. This design therefore separates and coordinates two therapeutic triggers, with tumor acidity controlling copper activation and light irradiation controlling PDT activation. The resulting spatiotemporal regulation enhances tumor cell killing while reducing toxicity to normal tissues, offering a stronger rationale for PDT–cuproptosis synergy than the broader claim that both modalities simply generate ROS [184] (Fig. 6A). One of the more interesting aspects of this combination is its potential spatial complementarity within heterogeneous tumors. PDT is impaired by severe hypoxia, whereas the effect of hypoxia on cuproptosis is context dependent because HIF-1α-driven glycolysis can also reduce mitochondrial pathway competence. This suggests complementary activity across heterogeneous tumor regions, with PDT favoring better oxygenated peripheral areas and cuproptosis remaining active in less oxygen-rich but mitochondria-competent subpopulations. Although this possibility still requires further experimental validation, it provides a biologically grounded framework for combining photodynamic oxidative injury with copper-dependent mitochondrial proteotoxic stress.

#### 4.1.4 Cuproptosis combined with sonodynamic and radiation therapy

For deep-seated tumors, ultrasound and ionizing radiation offer greater tissue penetration than near-infrared light, although the clinical maturity of sonodynamic therapy remains limited [[215], [216], [217], [218]]. Sonodynamic therapy (SDT) generates ROS through ultrasound-triggered cavitation-associated processes and can therefore amplify copper-mediated mitochondrial injury in tissues beyond the penetration depth of optical irradiation [159,219]. Recent nanoplatforms have begun to align this deep-tissue activation with cuproptosis. Ultrasound-triggered polymeric nanoparticles were designed to release copper ions and activate ROS production under ultrasound, thereby inducing cuproptosis and enhancing immunogenic SDT. This strategy links ultrasound-controlled oxidative stress with copper-dependent mitochondrial damage and antitumor immune activation [185] (Fig. 6B). A dual-responsive biomimetic CytoNano system further advanced this concept by combining copper nitride nanoparticles with oxygen-enriched components and membrane-based tumor targeting, enabling precision mitochondrial intervention through simultaneous SDT activation and cuproptosis induction [186]. In glioblastoma, carrier-free Ce6@Cu nano-sonosensitizers used the coordination between Cu^2+^ and chlorin e6 to create a self-assembled system in which ultrasound-amplified ROS generation, GSH depletion, ferroptosis and cuproptosis converged within tumor cells [187]. Cu-crosslinked near-infrared sonosensitizers provided another tumor-microenvironment-activated design, in which copper crosslinking enabled visualized in situ SDT, CDT and cuproptosis, while also supporting immune activation [188]. Together, these studies suggest that SDT–cuproptosis combinations are most compelling when ultrasound does more than generate ROS. In optimized systems, ultrasound also controls copper activation, improves deep-tissue precision, relieves hypoxia or antioxidant buffering, and converts local copper-driven tumor injury into immunogenic therapy.

Radiation therapy provides another clinically relevant route for coupling externally controlled energy deposition with cuproptosis. Copper can contribute to radiosensitization through redox cycling and, in appropriately designed systems, through enhanced local oxidative stress, whereas radiation itself can generate ROS, damage mitochondria and induce immunogenic stress. Functional nanocomposites based on Lipo-Ele@CuO_2_ exemplify this concept by combining a copper peroxide source with elesclomol-mediated mitochondrial copper delivery. This design supplies copper and H_2_O_2_, enhances mitochondrial copper stress, triggers cuproptosis and sensitizes tumors to radiotherapy [89]. A recent study reported that radiotherapy can increase mitochondrial copper stress and sensitize tumors to cuproptosis, but the generality of this mechanism remains to be established. Radiation was shown to increase mitochondrial copper accumulation, upregulate CTR1, deplete mitochondrial GSH, reduce lipoylated proteins and Fe–S cluster proteins, and synergize with cuproptosis inducers to overcome tumor radioresistance [189]. These findings shift the radiotherapy (RT)–cuproptosis relationship from a materials-based radiosensitization concept to a broader biological framework in which radiation itself may create a mitochondrial state permissive for copper-dependent cell death. Copper-enriched black phosphorus nanoplatforms further extend this principle by reprogramming the tumor microenvironment and enhancing low-dose radioimmunotherapy through cuproptosis sensitization, immunogenic cell death and immune remodeling [190] (Fig. 6C). Stimuli-responsive copper-iodide nanoparticles add another layer of external control by combining low-dose X-ray-induced photodynamic effects with copper release and enhanced cuproptosis, suggesting that radiation-triggered copper nanoplatforms can integrate radiotherapy, ROS amplification, and cuproptotic injury within a single system [191].

These studies collectively indicate that SDT and RT are not merely substitutes for optical activation, but distinct external-energy modalities that can expand cuproptosis-based nanomedicine toward deeper or more clinically challenging tumors. SDT offers spatially controllable deep-tissue ROS generation and can be coupled with copper release, oxygen modulation, ferroptosis, or immunogenic activation. RT provides an already established clinical treatment platform and may directly remodel mitochondrial copper handling and cuproptosis susceptibility. Nevertheless, several issues require careful clarification before these strategies can be considered translationally mature. Ultrasound dose, acoustic parameters, oxygen dependence and sonosensitizer degradation need to be standardized in SDT–cuproptosis systems. For RT–cuproptosis combinations, radiosensitization, radiation-induced ROS injury and canonical cuproptosis should be mechanistically separated using FDX1 dependence, lipoylated protein aggregation, Fe–S protein loss and copper-chelation rescue experiments. With further development, radiocopper-based nanoplatforms may become an especially attractive extension because copper radioisotopes could, in principle, unite imaging, radiation delivery and copper-dependent cell death within a single element. However, this theranostic concept still requires direct experimental validation in cuproptosis-specific nanomedicine models.

### 4.2 Cuproptosis and immunotherapy

The relationship between cuproptosis and antitumor immunity may be clinically important, although the underlying mechanisms remain incompletely defined. Beyond direct tumor-cell killing, cuproptotic stress may increase tumor immunogenicity, remodel suppressive microenvironments, and create conditions favorable for adaptive immune responses. Cuproptosis nanomedicine may therefore function not only as a local cytotoxic strategy but also as a platform for immune priming. However, immune-related conclusions must be interpreted according to the experimental model, because in vitro assays, immunodeficient xenografts, and immunocompetent tumor models provide fundamentally different levels of evidence.

**Fig. 7:**
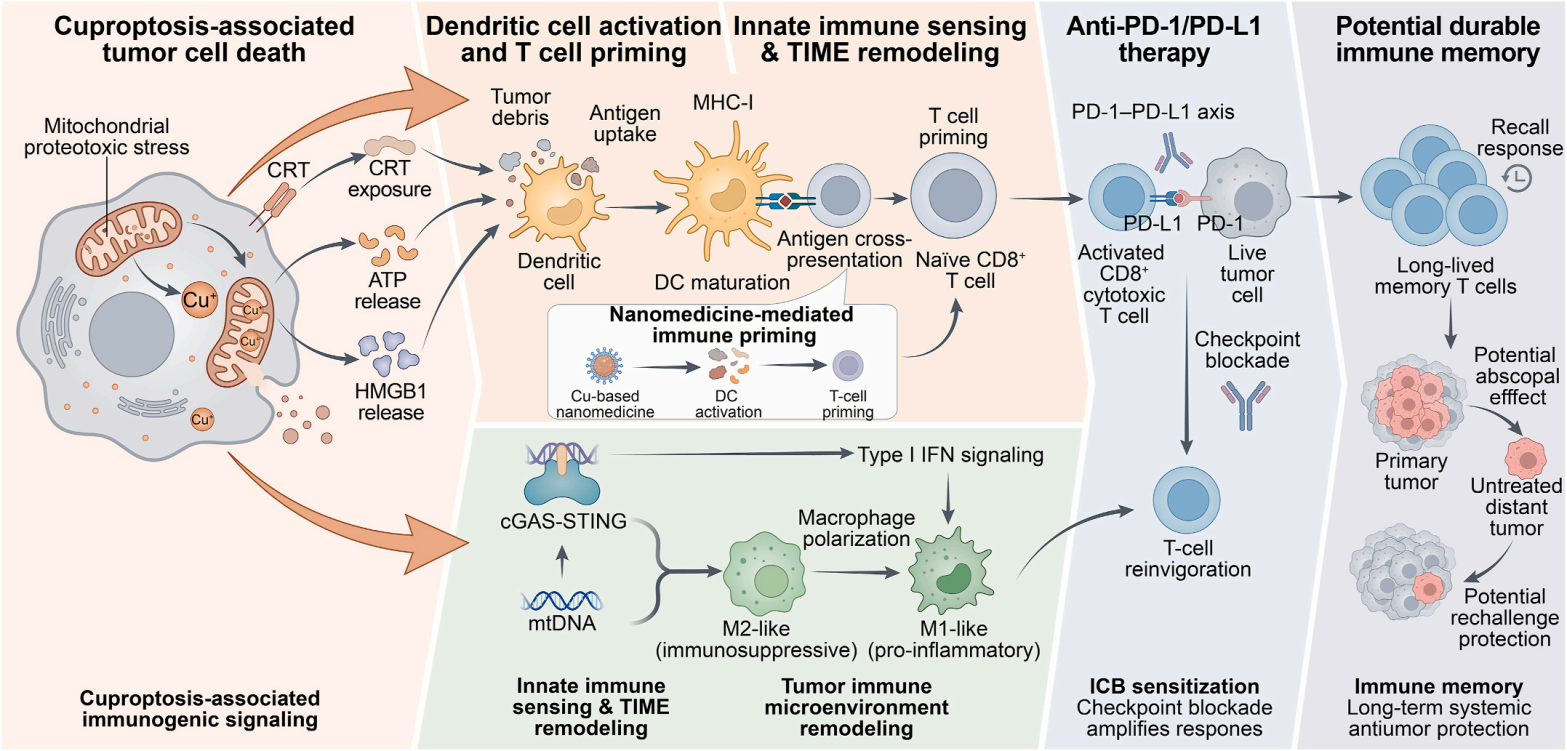
**Cuproptosis-associated immune activation and synergy with immune checkpoint blockade.** Cuproptosis-associated mitochondrial proteotoxic stress may promote calreticulin exposure, ATP release, and HMGB1 release, thereby facilitating tumor-antigen uptake, dendritic-cell maturation, MHC-I-mediated cross-presentation and CD8^+^ T-cell priming. Copper-based nanomedicines may also function as immune primers by enhancing dendritic-cell activation and T-cell responses. In parallel, mitochondrial DNA release can activate cGAS–STING signaling, induce type I interferon responses and remodel the tumor immune microenvironment, including macrophage polarization from an M2-like state toward an M1-like state. These immune effects may enhance T-cell reinvigoration and sensitize tumors to anti-PD-1 or anti-PD-L1 therapy. The resulting systemic immune response may contribute to control of untreated distant tumors and may support rechallenge protection or memory-like T-cell responses in selected preclinical models. **Note:** ATP, adenosine triphosphate; CD8^+^ T cell, cluster of differentiation 8-positive T cell; cGAS–STING, cyclic GMP–AMP synthase–stimulator of interferon genes; CRT, calreticulin; DC, dendritic cell; HMGB1, high mobility group box 1; ICB, immune checkpoint blockade; IFN, interferon; MHC-I, major histocompatibility complex class I; mtDNA, mitochondrial DNA; PD-1, programmed cell death protein 1; PD-L1, programmed death-ligand 1; TIME, tumor immune microenvironment.

**Fig. 8:**
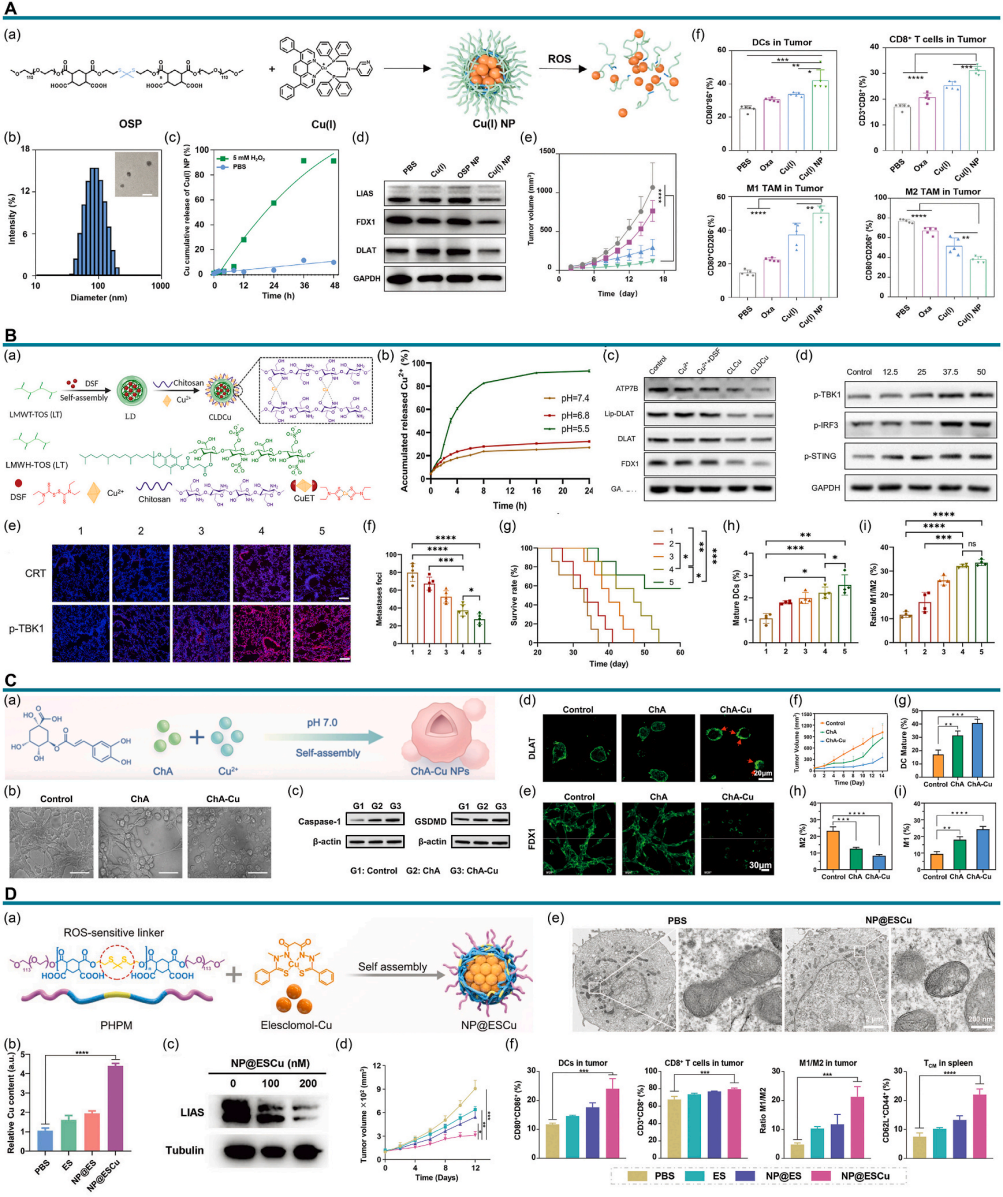
**Cuproptosis-driven immunotherapy and immune-cell engagement.** (**A**) Cu(I) NP induces stimulus-responsive cuproptosis and remodels immune-cell compartments. (a) Construction and ROS-responsive release scheme. (b) Particle size distribution and TEM image. (c) Copper release profile. (d) LIAS, FDX1, and DLAT expression. (e) Tumor growth curves. (f) DC maturation, CD8^+^ T-cell infiltration, and M1/M2 macrophage polarization. Reproduced from Ref. [112] under a Creative Commons license. (**B**) CLDCu integrates inhalable delivery, cuproptosis and cGAS–STING activation for lung metastasis immunotherapy. (a) Schematic design of CLDCu. (b) Cu^2+^ release under different pH conditions. (c) ATP7B, lipoylated DLAT, DLAT and FDX1 expression. (d) p-TBK1, p-IRF3 and p-STING expression. (e) CRT and p-TBK1 staining. (f, g) Lung metastasis and survival analysis. (h, i) DC maturation and M1/M2 macrophage ratio. Reproduced from Ref. [79] under a Creative Commons license. (**C**) ChA-Cu nanoparticles induce pyroptosis and cuproptosis to amplify antitumor immunity. (a) Self-assembly scheme. (b) Pyroptosis-associated cell morphology. (c) Caspase-1 and GSDMD expression. (d, e) DLAT and FDX1 staining. (f) Tumor growth curves. (g–i) DC maturation and macrophage polarization. Reproduced with permission [192]. Copyright 2025, Elsevier. (**D**) NP@ESCu co-delivers elesclomol and copper through a ROS-responsive nanoplatform to induce cuproptosis and enhance anti-PD-L1-mediated immunotherapy. (a) Self-assembly scheme of NP@ESCu. (b) Intracellular copper accumulation. (c) LIAS expression. (d) Tumor growth curves. (e) TEM images showing mitochondrial damage. (f) DC maturation, CD8^+^ T-cell infiltration, M1/M2 macrophage ratio and central memory T cells. Reproduced with permission [19]. Copyright 2023, Wiley. **Note:** Cu, copper; Cu(I), cuprous copper; NP, nanoparticle; ROS, reactive oxygen species; TEM, transmission electron microscopy; LIAS, lipoyl synthase; FDX1, ferredoxin 1; DLAT, dihydrolipoamide S-acetyltransferase; DC, dendritic cell; CD8^+^ T cell, cluster of differentiation 8-positive T cell; M1/M2, classically activated/pro-inflammatory macrophages and alternatively activated/immunosuppressive macrophages; CLDCu, inhalable cuproptosis-inducing nanodevice; cGAS, cyclic GMP-AMP synthase; STING, stimulator of interferon genes; Cu^2+^, cupric copper; ATP7B, ATPase copper transporting beta; p-TBK1, phosphorylated TANK-binding kinase 1; p-IRF3, phosphorylated interferon regulatory factor 3; p-STING, phosphorylated stimulator of interferon genes; CRT, calreticulin; ChA-Cu, chlorogenic acid-copper nanoparticles; GSDMD, gasdermin D; NP@ESCu, elesclomol-copper-loaded ROS-responsive nanoparticles; anti-PD-L1, anti-programmed death-ligand 1 antibody.

Immunogenic cell death is defined not simply by cell death, but by coordinated danger signaling that promotes antigen uptake, dendritic-cell maturation, cross-presentation, and T-cell priming [18,220,221]. Calreticulin exposure, ATP secretion, and HMGB1 release are widely used hallmarks of this process [[222], [223], [224]]. Copper ionophores and copper-based nanoparticles can induce these responses in tumor cells [19,86,225], suggesting that cuproptotic stress may possess immunogenic potential. In vitro measurements of calreticulin exposure, ATP secretion, HMGB1 release, mitochondrial DNA leakage, and cGAS–STING-related signaling are useful for identifying tumor-cell-intrinsic immunogenicity, but they do not establish functional antitumor immunity (Fig. 7). Stimulus-responsive Cu(I)-complex nanoparticles provide mechanistic support for this concept by increasing mitochondrial copper stress, promoting lipoylated DLAT aggregation, and enhancing antitumor immune responses [112] (Fig. 8A), while ultrasound-triggered polymeric nanoparticles couple spatially controlled copper release with immunogenic sonodynamic therapy [185] (Fig. 6B). Nevertheless, DAMP release should be interpreted as evidence of immunogenic potential rather than definitive proof of immunogenic cell death, particularly because many copper-based platforms activate multiple death pathways.

Cuproptosis-associated immune effects may arise through two overlapping routes. The first involves antigen and DAMP release from dying tumor cells, whereas the second reflects copper- and mitochondria-dependent signaling within the tumor microenvironment. Copper can influence macrophage polarization, checkpoint regulation, and innate immune activation [[226], [227], [228]], while mitochondrial injury may release mitochondrial DNA and activate the cGAS–STING pathway, leading to type I interferon signaling and antigen-presenting-cell activation [76,229,230]. Immunodeficient xenografts can support conclusions regarding nanoparticle delivery, tumor growth inhibition, pharmacodynamic cuproptosis markers, and local innate inflammation, but they cannot establish adaptive T-cell immunity or checkpoint sensitization. In contrast, immunocompetent syngeneic, orthotopic, metastatic, bilateral tumor, vaccination, and rechallenge models provide stronger functional evidence. The inhalable CLDCu nanoformulation promoted cGAS–STING signaling, dendritic-cell maturation, and antitumor immunity in lung metastasis models, particularly when combined with anti-PD-L1 treatment [79] (Fig. 8B). Self-assembled copper–chlorogenic acid nanoparticles further expanded this framework by combining cuproptosis with pyroptosis, thereby integrating mitochondrial proteotoxic stress with inflammatory signaling to enhance immune activation [192] (Fig. 8C). Future studies should determine whether cGAS–STING signaling originates primarily in tumor cells, myeloid cells, or both, using tumor-cell-specific pathway inhibition, cGAS- or STING-deficient models, and immune-cell-resolved analyses.

Immune checkpoint blockade is especially relevant because cuproptosis-associated inflammation may promote antigen presentation without being sufficient to sustain effector T-cell activity [[231], [232], [233]]. In this setting, cuproptosis may act as an immune-conditioning event, whereas checkpoint blockade reinforces and prolongs the adaptive response. ROS-responsive elesclomol–copper nanoparticles enhanced anti-PD-L1 efficacy in preclinical tumor models [19] (Fig. 8D), and CuP/Er nanoparticles that jointly induced ferroptosis and cuproptosis increased tumor-cell killing, T-cell proliferation and infiltration, and responsiveness to checkpoint blockade [193]. The inhalable CLDCu platform similarly combined cuproptosis, cGAS–STING activation, and anti-PD-L1 treatment [79] (Fig. 8B). These findings support a model in which cuproptotic stress shifts poorly inflamed tumors toward a more immune-permissive state. Claims of checkpoint sensitization should nevertheless be supported by spatially resolved CD8^+^ T-cell infiltration and activation, dendritic-cell maturation, myeloid-cell states, type I interferon signaling, and functional dependence on CD8^+^ T cells, cGAS, or STING. Tumor growth inhibition after combination treatment alone is insufficient to prove that cuproptosis directly mediates checkpoint sensitization.

Cuproptosis nanomedicine may also be explored as an in situ vaccination strategy, although current evidence remains insufficient to establish vaccine-like activity. Cuproptotic injury could provide tumor antigens and endogenous danger signals, while co-delivered STING agonists, toll-like receptor agonists, or selected neoantigens may strengthen adaptive priming [229,[234], [235], [236], [237]]. This approach may be particularly relevant in neoadjuvant and minimal-residual-disease settings, where durable systemic surveillance is as important as local tumor control. Current studies have reported abscopal effects, resistance to tumor rechallenge, and increases in memory T-cell populations after cuproptosis-oriented therapy, particularly when combined with checkpoint blockade [19,84,190,[235], [236], [237]]. However, the evidence remains heterogeneous and does not yet support the conclusion that cuproptosis consistently induces durable immune memory. At present, cuproptosis is better regarded as a potential immune-priming mechanism than as an established driver of long-term protective immunity. Future studies should distinguish tumor-cell-intrinsic immunogenic markers, pharmacodynamic findings in xenografts, and functional immune evidence from immunocompetent models, while incorporating antigen-specific CD8^+^ T-cell responses, effector-memory and central-memory T-cell populations, contralateral tumor control, tumor rechallenge resistance, long-term relapse monitoring, and immune-cell depletion.

### 4.3 Biochemical combinations and rational design rules

Biochemical combinations influence the intracellular conditions that determine whether cuproptosis can proceed efficiently. These strategies include chemotherapy, starvation therapy, gene-regulatory strategies, and the deliberate use of crosstalk with other regulated cell-death programs [[238], [239], [240], [241]]. Their value lies not in adding further external damage, but in removing intracellular barriers that would otherwise restrict effective cuproptosis. They are also mechanistically informative, because they help identify the intracellular factors that most strongly limit cuproptosis in real tumors.

**Fig. 9:**
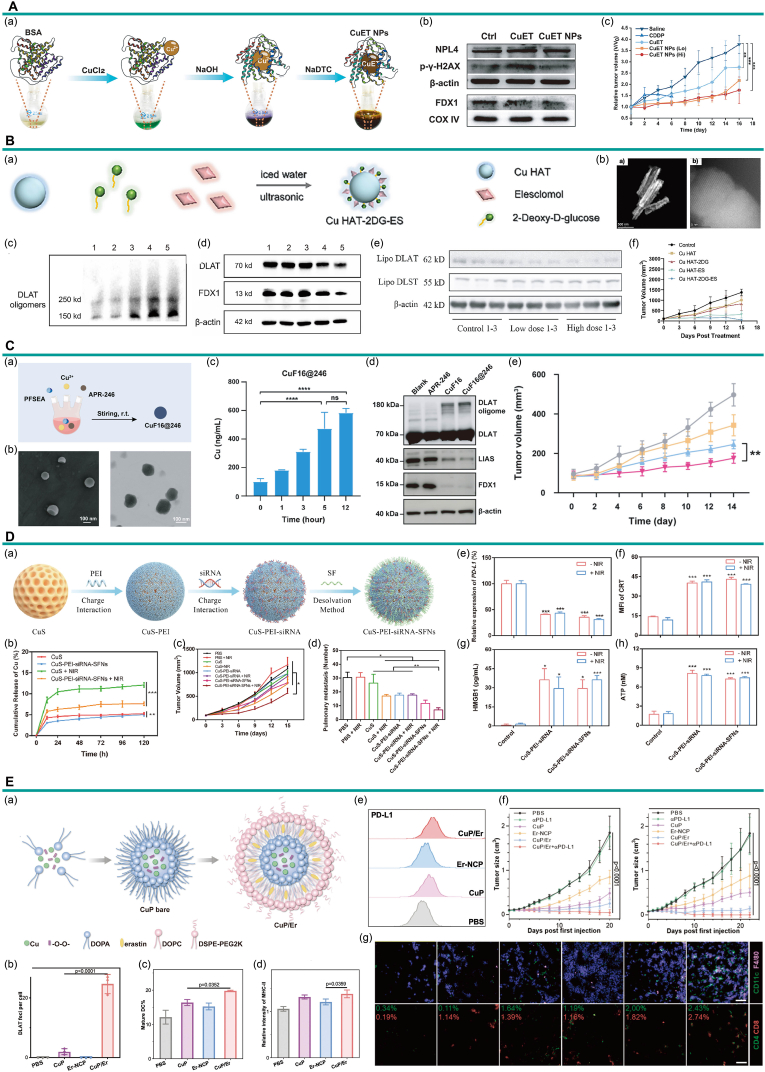
**Biochemical combinations and regulated cell-death crosstalk based on cuproptosis.** (**A**) CuET nanoparticles reverse cisplatin resistance through glutathione-resistant cuproptosis. (a) Preparation scheme of BSA-stabilized CuET nanoparticles. (b) NPL4, γH2AX and FDX1 expression. (c) Tumor growth curves in a cisplatin-resistant model. Reproduced with permission [201]. Copyright 2022, Royal Society of Chemistry. (**B**) Cu HAT-2-DG-ES nanotubes combine copper delivery, elesclomol-mediated copper transport, and 2-DG-induced starvation therapy. (a) Preparation scheme of Cu HAT-2-DG-ES. (b) Nanotube morphology. (c) DLAT oligomerization. (d) DLAT and FDX1 expression. (e) Lipoylated DLAT and DLST expression. (f) Tumor growth curves. Reproduced from Ref. [194] under a Creative Commons license. (**C**) CuF16@246 couples copper delivery with APR-246-mediated p53 reactivation to promote metabolic reprogramming and cuproptosis. (a) Construction scheme. (b) SEM and TEM images. (c) Intracellular Cu levels after incubation with CuF16@246. (d) DLAT oligomerization, LIAS, and FDX1 expression. (e) Tumor growth curves. Reproduced with permission [196]. Copyright 2025, Elsevier. (**D**) CuS-PEI-siRNA-SFNs combine cuproptosis with PD-L1 siRNA-mediated immune checkpoint silencing. (a) Layer-by-layer construction scheme. (b) Copper release profile. (c) Tumor growth curves. (d) Pulmonary metastasis quantification. (e) PD-L1 expression. (f–h) CRT, HMGB1 and ATP levels indicating immunogenic cell death. Reproduced from Ref. [202] under a Creative Commons license. (**E**) CuP/Er nanoparticles synergize ferroptosis and cuproptosis to enhance immunotherapy. (a) Construction scheme. (b) DLAT puncta quantification. (c, d) Mature DCs and MHC-II expression. (e) PD-L1 expression. (f) Tumor growth curves with or without anti-PD-L1. (g) Immune-cell infiltration in tumor tissues. Reproduced from Ref. [193] under a Creative Commons license. **Note:** Cu, copper; CuET, copper diethyldithiocarbamate; BSA, bovine serum albumin; GSH, glutathione; NPL4, nuclear protein localization protein 4 homolog; γH2AX, phosphorylated H2A histone family member X; FDX1, ferredoxin 1; Cu HAT, copper-based hollow amorphous tannic acid nanotube; 2-DG, 2-deoxy-D-glucose; ES, elesclomol; DLAT, dihydrolipoamide S-acetyltransferase; DLST, dihydrolipoamide S-succinyltransferase; CuF16@246, copper-containing nanocoordination polymer loaded with APR-246; SEM, scanning electron microscopy; TEM, transmission electron microscopy; LIAS, lipoyl synthase; PD-L1, programmed death-ligand 1; siRNA, small interfering RNA; CuS, copper sulfide; PEI, polyethyleneimine; SFNs, silk fibroin nanoparticles; CRT, calreticulin; HMGB1, high mobility group box 1; ATP, adenosine triphosphate; ICD, immunogenic cell death; CuP/Er, copper nanoparticle loaded with erastin; DC, dendritic cell; MHC-II, major histocompatibility complex class II; anti-PD-L1, anti-programmed death-ligand 1 antibody.

Chemotherapy is most useful in cuproptosis-based combinations when it changes the cellular state in ways that favor cuproptotic execution. One mechanism involves transport, because copper and platinum drugs share parts of the same uptake and efflux machinery, including CTR1 and ATP7A or ATP7B [66,67]. A similar effect may arise through stress adaptation, as several chemotherapeutics perturb mitochondria and shift surviving cells toward greater oxidative dependence [242]. In this way, chemotherapy may enhance cuproptosis not only through parallel cytotoxicity, but also by altering tumor metabolism, redox buffering or drug-transport pathways in ways that increase susceptibility to copper stress. DSF–copper chemistry provides an important translational and nanomedicine entry point for this strategy. Stimuli-responsive nanoplatforms that form DSF–copper complexes in situ can mitigate the instability and pharmacokinetic limitations of free DSF while enabling tumor-responsive CuET formation and coordinated chemo–copper therapy [197]. Clinically, the combination of DSF and copper with concurrent radiotherapy and temozolomide in newly diagnosed glioblastoma provides a relevant example of integrating copper-dependent cytotoxicity with standard-of-care chemoradiotherapy, although this approach should be interpreted as copper-directed combination therapy rather than definitive proof of canonical cuproptosis in patients [198]. Platinum-based chemotherapy offers another mechanistically coherent partner for copper-based nanomedicine. Copper peroxide and cisplatin co-loaded silica nanoparticles were designed as a trinity strategy that integrates cuproptosis, chemotherapy and chemodynamic therapy [199]. In this system, CuO_2_ supplies copper and H_2_O_2_, consumes GSH, relieves tumor hypoxia and acidosis, and helps block cisplatin efflux, while cisplatin adds DNA damage and ROS burden. The resulting combination promotes DLAT oligomerization and FDX1-related cuproptosis events, enhances CDT and improves cisplatin sensitization [199]. Copper-coordinated doxorubicin nanomedicine provides a complementary example in lung cancer, where a Cu^2+^-coordinated lignosulfonate/doxorubicin complex responds to acidic and GSH-rich conditions to release both copper and doxorubicin [200]. The copper component induces cuproptosis-associated mitochondrial injury, whereas doxorubicin promotes apoptotic chemotherapy and contributes H_2_O_2_ for Fenton-like oxidative amplification, creating a coupled cuproptosis–apoptosis–redox injury program [200]. For drug-resistant tumors, glutathione-resistant CuET nanomedicine illustrates how cuproptosis can be used to bypass antioxidant-mediated chemotherapy resistance. Because CuET is relatively resistant to GSH inactivation, albumin-stabilized CuET nanoparticles retain copper-based cytotoxicity in high-GSH cisplatin-resistant non-small cell lung cancer cells and reverse cisplatin resistance through cuproptosis-associated mechanisms [201] (Fig. 9A). Overall, chemotherapy appears most valuable in cuproptosis combinations when it changes transport, metabolism or redox state in ways that increase susceptibility to mitochondrial copper injury. These examples suggest that the strongest chemo–cuproptosis combinations are not simple co-delivery systems, but coordinated designs in which chemotherapy, copper release, GSH modulation and mitochondrial stress reinforce one another.

Starvation-based strategies are relevant to cuproptosis because they alter tumor metabolic dependence and weaken the metabolic states that protect cancer cells from mitochondrial copper stress. GOx-mediated glucose depletion can generate H_2_O_2_ and impose metabolic stress, which may increase cuproptosis susceptibility in selected tumor contexts [148,149,195]. Pharmacological glycolysis inhibitors such as 2-deoxy-D-glucose (2-DG) may achieve a related effect with potentially simpler chemistry [146]. This design logic has been further supported by copper-based nanotubes that integrate starvation therapy with cuproptosis for synergistic cancer treatment [194]. In this system, the nanotube architecture serves as a copper-containing therapeutic platform that perturbs tumor metabolic homeostasis while inducing copper stress, thereby linking nutrient deprivation with cuproptosis induction. By enhancing starvation pressure and copper-dependent mitochondrial injury within the same nanoplatform, this strategy illustrates how metabolic restriction can be converted from a standalone cytostatic intervention into a sensitizing step for copper-triggered cell death (Fig. 9B). Beyond glucose deprivation, lactate metabolism has emerged as another actionable metabolic node for cuproptosis sensitization. In a biomimetic lactate oxidase-loaded copper nanoregulator, lactate depletion reshaped the metabolic and redox state of clear cell renal cell carcinoma cells, while lactate oxidase-generated H_2_O_2_ amplified ROS production and copper-dependent mitochondrial injury [183]. This design links lactate clearance, ROS storm formation, cuproptosis and metalloimmunotherapy, suggesting that tumor-associated lactate can be converted from an immunometabolic barrier into a substrate for copper-based metabolic amplification. Metabolic reprogramming can also be coupled to tumor-suppressor restoration. A dual-functional copper nanoplatform was reported to reactivate p53 while potentiating cuproptosis, thereby connecting copper stress with p53-dependent metabolic rewiring [196] (Fig. 9C). Because p53 can influence glycolysis, mitochondrial respiration, redox balance and cell-death susceptibility, this strategy broadens the metabolic logic of cuproptosis beyond nutrient depletion alone and suggests that restoring tumor-suppressive metabolic control may increase the vulnerability of cancer cells to copper-triggered proteotoxic stress. Such approaches are most effective when they move tumors away from a glycolysis-protected state and toward one in which mitochondrial respiration, lipoylated TCA-cycle proteins and copper-sensitive proteotoxic stress become more consequential.

Gene-regulatory strategies address cuproptosis resistance more directly by targeting the pathways that restrict copper retention, preserve antioxidant buffering, or suppress antitumor immunity. Rather than simply increasing the delivered copper dose, recent studies suggest that therapeutic resistance can be reprogrammed at the levels of copper transport and immune checkpoints. Polydopamine-based copper-deposition nanostructures have been used to promote cuproptosis by catalytically inhibiting copper exporters in tumor cells, thereby increasing intracellular copper retention and enhancing cancer immunotherapy [117]. Similarly, copper-homeostasis-regulating nanoplatforms have been shown to promote tumor-cell cuproptosis and enhance breast cancer immunotherapy by shifting the balance between copper influx, efflux, and intracellular copper accumulation [118]. This concept is further supported by mechanistic evidence that ATP7A-dependent copper metabolic homeostasis sustains mutant KRAS-driven lung cancer progression, whereas targeting ATP7A disrupts copper handling, induces cuproptosis and suppresses tumor growth [119]. In parallel, gene-silencing approaches can be used to reduce immune escape rather than directly modulate copper transport. A layer-by-layer silk fibroin nanoplatform loaded with PD-L1 siRNA used a copper sulfide–polyethylenimine (CuS-PEI) core to combine cuproptosis with photothermal and chemodynamic therapy, while PD-L1 silencing enhanced antitumor immune activation and suppressed metastatic breast cancer [202] (Fig. 9D). Together, these studies support a broader design rule in which nucleic acid or gene-regulatory modules are paired with copper nanomedicine to block copper efflux, weaken resistance pathways, or relieve immunosuppression. Their appeal lies in targeting resistance at its source rather than attempting to overcome it with progressively higher copper doses. However, direct evidence for some proposed routes, including solute carrier family 7 member 11/xCT cystine–glutamate antiporter (SLC7A11/xCT) siRNA-mediated GSH depletion or FDX1 restoration through mRNA delivery in copper nanomedicine, remains less mature than the evidence for copper-exporter regulation and PD-L1 siRNA delivery. The main translational limitations remain efficient in vivo nucleic-acid delivery, tumor-selective uptake, endosomal escape, long-term safety and the manufacturing and regulatory complexity of multicomponent gene–metal nanoplatforms.

Crosstalk between cuproptosis and other regulated cell death programs is receiving increasing attention in cuproptosis nanomedicine. Among these interactions, the cuproptosis–ferroptosis axis is particularly compelling because the two pathways share several biochemical vulnerabilities, including glutathione dependence, redox-active metal chemistry, mitochondrial stress, and immunogenic consequences [73,74,[243], [244], [245], [246]]. Recent nanoplatform studies have begun to exploit this convergence more explicitly. Carrier-free Ce6@Cu nano-sonosensitizers use coordination-driven self-assembly between chlorin e6 and copper to integrate sonodynamic therapy with copper-dependent mitochondrial injury and ferroptosis in glioblastoma [187]. Under ultrasound irradiation, Ce6-mediated ROS production amplifies oxidative stress, whereas copper contributes to cuproptosis-associated mitochondrial damage and disrupts redox homeostasis. The resulting combination promotes both lipid peroxidation and copper-triggered proteotoxic stress, providing a rationale for sonodynamic-amplified cuproptosis–ferroptosis therapy in a tumor type where deep-tissue activation is especially relevant. This concept has been further developed in immunotherapy-oriented nanoplatforms that deliberately couple ferroptosis with cuproptosis to enhance antitumor immune activation [193]. Here, ferroptotic lipid peroxidation and cuproptotic mitochondrial proteotoxic stress converge to increase tumor cell immunogenicity, enhance immune activation and improve the response to immunotherapy [193] (Fig. 9E). Together, these studies suggest that the cuproptosis–ferroptosis combination is most convincing when both pathways are linked through shared GSH depletion, metal-catalyzed oxidative damage and mitochondrial vulnerability, rather than being induced as unrelated parallel stresses. A possible link to pyroptosis is also suggested by mitochondrial injury, mtDNA leakage and inflammasome engagement, which together may connect cuproptosis-associated stress with inflammatory signaling [81,82,104,192]. If confirmed rigorously, this would support a mechanistic bridge between mitochondrial proteotoxicity and interleukin-1 beta (IL-1β) or interleukin-18 (IL-18) release, with possible implications for immune recruitment. Even so, the broader field still needs a clearer distinction between true sequential crosstalk and parallel activation of mixed death programs in different tumor cell subsets. For cuproptosis–ferroptosis systems, this requires paired rescue strategies, including copper chelation or FDX1/lipoylation-pathway manipulation for cuproptosis, and ferrostatin-1, liproxstatin-1, glutathione peroxidase 4 (GPX4), or acyl-CoA synthetase long-chain family member 4 (ACSL4)-based validation for ferroptosis. Without such experiments, the term “synergy” risks describing phenotypic co-occurrence rather than mechanistically connected cell-death crosstalk.

The design of cuproptosis-based combination strategies should be guided by mechanism rather than by the simple accumulation of therapeutic modules. Each additional component should address a defined biological constraint that limits cuproptosis induction, propagation or antitumor efficacy. In this context, a partner therapy is valuable only when it overcomes a barrier that cuproptosis alone cannot resolve, rather than merely imposing another layer of cellular stress. Therapeutic sequencing is equally important, as the order of intervention can markedly influence efficacy. For example, intracellular copper buffering may need to be attenuated before copper release, whereas immune priming may need to occur before checkpoint blockade [90,143]. Effective synergy also requires spatial coordination, with both components reaching the same target cells and, ideally, the same relevant intracellular site [25]. Rational combinations should widen rather than narrow the therapeutic window [25,129]. The choice of partner therapy should also be matched to the biological context of each tumor type. Future progress will depend on identifying the smallest mechanism-matched combination that can sustain cuproptosis and produce durable tumor control.

## 5 Translational landscape

**Fig. 10:**
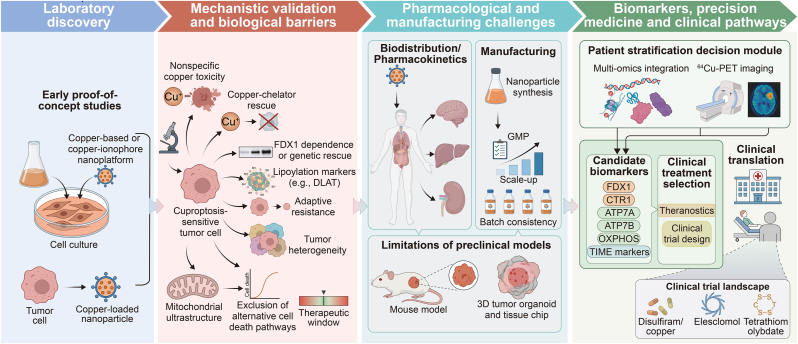
**Translational roadmap and clinical challenges for cuproptosis nanomedicine.** Translation from laboratory discovery to clinical application requires coordinated progress in mechanistic validation, pharmacology, manufacturing, biomarker development and precision medicine. Early proof-of-concept studies use copper-based or copper-ionophore nanoplatforms to identify cuproptosis-sensitive tumor models. Mechanistic validation should distinguish bona fide cuproptosis from nonspecific copper toxicity through copper-chelator rescue, FDX1 and protein-lipoylation analyses, mitochondrial ultrastructure, exclusion of alternative death pathways, and assessment of adaptive resistance, tumor heterogeneity and the therapeutic window. Pharmacological development requires characterization of biodistribution, pharmacokinetics, organ accumulation and preclinical model limitations, together with scalable nanoparticle synthesis, GMP-compliant manufacturing and batch-to-batch consistency. Biomarker-guided translation may integrate multi-omics, ^64^Cu-PET imaging, FDX1, CTR1, ATP7A/ATP7B, OXPHOS and tumor immune microenvironment markers to support theranostic treatment selection and rational clinical trial design. Disulfiram–copper, elesclomol and tetrathiomolybdate provide clinical precedents for copper-modulating therapy, although they do not represent clinically validated cuproptosis nanomedicines. **Note:**^64^Cu-PET, copper-64 positron emission tomography; ATP7A/ATP7B, ATPase copper transporting alpha/beta; CTR1, copper transporter 1; FDX1, ferredoxin 1; GMP, Good Manufacturing Practice; OXPHOS, oxidative phosphorylation; TIME, tumor immune microenvironment.

The molecular basis of cuproptosis, the principles of nanoplatform design, and the logic of combination therapy together provide a strong foundation for translational development. However, a strong preclinical rationale is not sufficient to establish a clinically viable therapeutic platform. In cancer nanomedicine, only a limited number of formulations have achieved regulatory approval, and their success has often depended more on improved pharmacokinetics, tolerability, and manufacturability than on increasingly complex targeting strategies [24,152,247]. Cuproptosis nanomedicine faces many of the same barriers that have slowed the broader nanomedicine field, including difficulty in scale-up, incomplete pharmacokinetic characterization, limited predictive value of standard preclinical models, and regulatory ambiguity. It also faces copper-specific challenges, including difficulty distinguishing bona fide cuproptosis from nonspecific copper toxicity, a poorly defined therapeutic window for copper-based interventions, and a lack of clinically validated biomarkers for patient selection [28,31,131,248]. The key challenge is not whether cuproptosis can be induced experimentally, but whether it can be triggered selectively, reproducibly and measurably in patients. Progress will require an integrated framework combining mechanistic validation, pharmacological and manufacturing feasibility, biomarker-guided patient selection, and rational clinical trial design (Fig. 10).

### 5.1 Mechanistic validation and biological barriers

Clinical translation first requires convincing evidence that the intended pathway is engaged in biologically relevant tumor models. A central issue is what should count as convincing evidence that cuproptosis, rather than generic copper injury, is being engaged in a therapeutically meaningful way. This distinction shapes the interpretation of preclinical efficacy, biomarker selection, and the definition of target engagement in early-phase clinical studies. At present, the main biological barriers extend beyond insufficient efficacy and include limited mechanistic rigor, insufficient distinction between specific and nonspecific copper injury, limited understanding of heterogeneity-driven escape, and poor definition of the safety window for therapeutic copper perturbation [28,31,248].

#### 5.1.1 The cuproptosis validation gap

A major obstacle in the field is the lack of rigorous validation for cuproptosis, as many claims still rely on limited mechanistic evidence. In many reports, cuproptosis is inferred when a copper-containing nanoplatform induces cell death and alters one or two pathway-associated proteins, such as DLAT or FDX1 [16,249]. However, this standard is insufficient because it risks conflating cuproptosis with broader copper-associated cytotoxicity and obscures the specific mechanistic contribution of nanoplatform design. From a translational perspective, this is a major weakness because regulatory and clinical development require target engagement to be demonstrated more rigorously [28,31,248]. Claims of cuproptosis should be supported by convergent biochemical, pharmacological, and genetic evidence, as described in the mechanistic validation criteria in Section 2.2 [10,12,13,16,60]. Where only one or two of these criteria are met, the findings are more appropriately described as copper-dependent cell death rather than definitive evidence of cuproptosis. A standardized validation checklist would help bring cuproptosis studies closer to the evidentiary standards used for other regulated cell death programs [31,248].

#### 5.1.2 Distinguishing cuproptosis from nonspecific copper toxicity

Distinguishing cuproptosis from broader copper toxicity remains an important translational challenge because it directly affects mechanistic interpretation, dose selection, and biomarker development. Copper is intrinsically reactive and, at sufficiently high concentrations, can generate ROS, oxidize proteins and lipids, damage DNA, disrupt membranes, and activate multiple stress responses simultaneously [2,40,151]. By contrast, cuproptosis is a more specific form of copper-dependent cell death that involves FDX1, lipoylation, and the Fe–S axis [10,36]. For translational development, copper dose needs to remain within a biologically meaningful range, because below this range, cuproptosis may be only incompletely engaged, whereas above it, nonspecific toxicity may dominate and mechanistic interpretation becomes unreliable [28,248]. Dose–response studies should pair pathway-specific readouts, such as DLAT oligomerization and Fe–S protein loss, with generic injury markers, including lactate dehydrogenase release, intracellular ROS accumulation and phosphorylated histone H2AX (γH2AX). Matched experiments in FDX1-deficient cells would further strengthen causal inference [10,16,249]. The challenge is even greater in vivo, where static immunohistochemistry may provide endpoint clues but cannot capture the dynamic onset of cuproptosis within living tumors. Cuproptosis-specific biosensors, mitochondrial copper reporters, and imaging-compatible readouts of Fe–S integrity therefore remain important technical gaps in the field. Without such tools, it remains difficult to distinguish true pathway engagement from copper overload in general [28,31].

#### 5.1.3 Tumor heterogeneity and adaptive resistance

Tumor heterogeneity remains a major translational obstacle, even when cuproptosis can be induced reliably [250,251]. Clinical tumors are metabolically heterogeneous, and oxidative phosphorylation (OXPHOS)-dependent and glycolysis-dependent populations often coexist under the influence of local oxygenation, nutrient gradients, and therapy-induced stress [145,252,253]. As a result, cuproptosis may effectively eliminate one tumor compartment while leaving glycolytic survivors capable of repopulating the lesion. These observations suggest that cuproptosis should not be developed on the assumption that tumor susceptibility is a binary property of the whole tumor mass. Rather, susceptibility is better understood as a dynamic ecological state within the tumor [28]. Adaptive resistance may further complicate the clinical translation of cuproptosis nanomedicine. Repeated or chronic copper exposure may promote several adaptive changes, including enhanced efflux through ATP7A or ATP7B, reinforcement of NRF2-associated thiol buffering, increased metallothionein expression, epigenetic suppression of FDX1, and a metabolic shift from OXPHOS toward glycolysis [17,47,141,145,242,254,255]. Although adaptive resistance remains underexplored experimentally, future cuproptosis nanomedicine will likely need to incorporate resistance-aware design, either by preventing these adaptations from emerging or by integrating biomarkers capable of detecting them early [28,31].

#### 5.1.4 Safety and toxicology considerations

Copper safety remains a major consideration in the translational development of cuproptosis-based nanomedicine [[256], [257], [258]]. Human disorders of copper metabolism demonstrate that the liver, nervous system, kidney and other organs are highly susceptible to disruption of copper homeostasis [[259], [260], [261], [262]]. Wilson disease provides a clinically relevant model of chronic copper dysregulation and demonstrates that persistent defects in copper trafficking can cause severe multi-organ injury. This clinical background is directly relevant to cuproptosis nanomedicine, for which toxicity should be interpreted in the context of established human copper pathophysiology [17,28]. Safety assessment should therefore extend beyond acute tolerability to include long-term systemic copper imbalance, especially after repeated dosing. Key parameters should include serum copper, ceruloplasmin concentration/activity, hepatic and renal copper burdens, urinary and biliary copper excretion, and delayed organ injury, which together can indicate whether therapeutic copper delivery perturbs whole-body copper homeostasis [17,[259], [260], [261]].

The liver is likely to remain the primary toxicological concern, owing to the extensive uptake of intravenously administered nanoparticles by the mononuclear phagocyte system and the central role of the liver in biliary copper handling [27,129,259,[263], [264], [265]]. Renal safety also requires careful evaluation because released copper species, small nanomaterial fragments, and excretion-related exposure may injure renal tubular cells or impair filtration. Quantitative biodistribution studies should distinguish intact nanoplatforms from released copper in blood, tumor, and major clearance organs [264,265]. CuO nanoparticles have also been associated with pulmonary inflammation, underscoring the need for route-specific exposure assessment and organ-resolved toxicological evaluation [266]. Neurological risk also warrants attention, particularly when tumor-associated inflammation, surgery or radiotherapy compromises the blood–brain barrier [260,267]. This concern is amplified by evidence that copper overload can impair neuronal mitochondrial function, alter mitochondrial dynamics and mitophagy, promote oxidative injury, and contribute to neuroinflammation and cognitive dysfunction [268].

Immune-related toxicity represents another underexplored safety dimension. Many cuproptosis nanomedicines are designed to induce immunogenic cell death, activate cGAS–STING signaling or sensitize tumors to immune-checkpoint blockade. If such immune activation is excessive or poorly localized, it may cause cytokine imbalance, complement activation, macrophage or dendritic-cell perturbation, and unintended inflammatory injury [[269], [270], [271], [272]]. This concern is consistent with nanotoxicology studies showing that nanomaterials can interact with immune cells and that copper oxide nanoparticles may alter lymphocyte function, cytokine production, and antioxidant defenses after subchronic exposure [[269], [270], [271]].

Because canonical cuproptosis depends on mitochondrial respiration, normal OXPHOS-dependent tissues, including the myocardium, neurons, renal tubular cells, hepatocytes, and skeletal muscle, may be vulnerable when copper release is insufficiently tumor-restricted [10,16,254,268]. Defining the therapeutic window for copper-based nanomedicine should therefore be a major preclinical priority rather than a routine component of toxicology testing. Preclinical development should define the maximum tolerated dose, dose-limiting toxicities and no-observed-adverse-effect level, while mechanistic studies distinguish systemic copper perturbation from organ-specific injury [28,131]. Future safety studies should incorporate repeated-dose toxicology, recovery-period assessments, inductively coupled plasma mass spectrometry (ICP-MS)-based tissue copper quantification, liver and kidney function tests, histopathology, neurobehavioral assessment, cardiac and mitochondrial toxicity markers, cytokine profiling, complement activation assays, immune-cell phenotyping, and direct comparisons between tumor cuproptosis and normal-tissue mitochondrial stress.

### 5.2 Pharmacological and manufacturing challenges

Even if cuproptosis is biologically valid and therapeutically meaningful, translation will still fail if the nanoplatform cannot be described reproducibly, manufactured consistently, or evaluated in models that predict human behavior with adequate fidelity. This reflects a broader lesson from nanomedicine: biological plausibility alone is insufficient unless formulation properties support rational dosing, reproducible manufacturing and regulatory evaluation [131,156,273,274].

#### 5.2.1 Pharmacokinetics of copper-based nanomedicine

The pharmacokinetic profile of copper-based nanomedicine remains insufficiently characterized. Many studies still report tumor copper content only at a terminal time point, without adequately characterizing circulation of the intact nanoplatform, copper release kinetics, organ distribution of liberated copper, or accumulation after repeated dosing [263]. This limitation has practical consequences because it hinders rational dose selection, obscures cumulative toxicity risk, and leaves clinical trial design without a sufficient quantitative basis. For cuproptosis nanomedicine, pharmacokinetics must distinguish the disposition of the intact carrier from the timing and location of bioavailable copper release [131]. Non-invasive imaging and spatial metal analysis may help address this limitation. For example, copper-64 positron emission tomography (^64^Cu-PET) can track whole-body distribution, whereas laser ablation–inductively coupled plasma mass spectrometry (LA-ICP-MS) can map regional tissue copper. Organelle-level localization requires complementary fractionation or organelle-resolved imaging [158,[275], [276], [277]]. Route of administration also influences translational feasibility. Although intravenous delivery remains the default in most studies, intratumoral injection and sustained-release hydrogels may be more practical for selected lesions because they reduce systemic copper exposure and may preserve immune-priming effects through in situ vaccination-like mechanisms [18,233,278,279]. More complete pharmacokinetic–pharmacodynamic (PK–PD) frameworks are therefore needed to link copper disposition directly to cuproptosis engagement and toxicity risk, rather than relying only on single-endpoint biodistribution data [131].

#### 5.2.2 Manufacturing scalability and quality control

The gap between laboratory synthesis and Good Manufacturing Practice (GMP)-compliant manufacturing remains a persistent obstacle in nanomedicine, and copper-based systems are likely to face the same challenge [24,152,263]. Inorganic copper nanoparticles may perform reproducibly on a small laboratory scale, yet scale-up can alter particle size, morphology, copper content, surface chemistry and release kinetics. A copper core, targeting ligand, redox-responsive polymer, mitochondria-directing element, and biomimetic shell may each serve a clear mechanistic purpose, but together they create multiple sources of batch failure and analytical uncertainty. This makes formulation realism especially important for translation [131]. Quality control presents a similar challenge, because clinically relevant translation depends on consistent characterization of multiple critical formulation attributes. These include particle size and dispersity, morphology, total copper content, copper oxidation state, release kinetics under multiple biologically relevant conditions, surface chemistry, endotoxin burden, sterility, storage stability, and batch-to-batch reproducibility [152,263]. Few published cuproptosis studies provide this degree of analytical characterization, which further supports a minimalist platform-design strategy. Platforms built from clinically familiar materials and minimal functional components are easier to produce and more likely to achieve regulatory-grade reproducibility [131,156,273].

#### 5.2.3 Limitations of current preclinical models

Current preclinical model systems remain poorly aligned with the biological features most relevant to the clinical translation of cuproptosis. In particular, subcutaneous xenografts in immunodeficient mice do not recapitulate the orthotopic microenvironment, metabolic heterogeneity, or intact antitumor immunity of human tumors [280,281]. This mismatch is especially important for cuproptosis, whose efficacy is shaped by metabolic heterogeneity and whose proposed benefits, particularly in combination with immunotherapy, depend on immune system participation. Model systems that exclude both intratumoral metabolic diversity and adaptive immunity are therefore poorly suited to the key translational questions in the field [31,131]. Another limitation is the widespread use of rapidly proliferating, relatively homogeneous cell line-derived tumors. Although these models are useful for proof-of-principle studies, they remain poor surrogates for the spatial and metabolic heterogeneity of the lesions in which cuproptosis would need to function clinically [252,281]. This may lead the field to overestimate uniform tumor susceptibility and underestimate the emergence of resistant subpopulations [28].

#### 5.2.4 Toward more relevant preclinical evaluation

A more credible preclinical pipeline should be built around complementary rather than uniform models. Syngeneic and orthotopic models are essential when the aim is to assess immune engagement, microenvironment remodeling, or combinations with checkpoint blockade [280,281]. Patient-derived xenograft (PDX) models are useful for preserving tumor heterogeneity, although their immunodeficient host setting limits immune interpretation [282]. Genetically engineered mouse models (GEMMs) offer a more physiologically relevant setting, but they require careful matching to genotypes and metabolic states relevant to cuproptosis [283]. Organoids and organ-on-chip systems can complement these in vivo models by providing more controlled settings for studying heterogeneity, vascular barriers, and delivery behavior [[284], [285], [286]]. Preclinical models should be selected according to the specific translational question they are intended to address, rather than for convenience or convention [131]. Safety evaluation will also require large-animal pharmacology and toxicology, particularly because the liver and nervous system are sensitive to copper dysregulation [259,263]. Given its systemic reactivity, cuproptosis nanomedicine is unlikely to be evaluated with the same degree of flexibility sometimes applied to less reactive payloads. Species-relevant copper toxicology will therefore become increasingly important as these platforms move closer to clinical translation [28,131].

### 5.3 Biomarkers, precision medicine, and clinical pathways

The metabolic and molecular heterogeneity of cuproptosis sensitivity makes clinical development in unselected patient populations especially risky [287]. The phase III elesclomol trial did not improve progression-free survival in an unselected melanoma population, although post hoc analyses suggested heterogeneity according to baseline lactate dehydrogenase [21,288]. For cuproptosis nanomedicine, biomarker-guided development is therefore likely to be essential for clinical success rather than an optional refinement [28,[289], [290], [291]].

Candidate biomarkers for cuproptosis-based therapy can be considered in relation to three broad aspects of tumor biology: pathway competence, copper handling, and metabolic state. Pathway competence is reflected by FDX1, DLAT, LIAS, LIPT1, and other indicators of lipoylation-dependent substrate availability [10,16,60,255]. Copper handling is reflected by CTR1 and ATP7A or ATP7B, which together influence copper uptake and efflux [47,66,292]. Metabolic state is reflected in the balance between oxidative phosphorylation and glycolysis, which may determine whether tumor cells retain the metabolic context required for cuproptosis [145,252,253]. Taken together, these considerations suggest that susceptibility is shaped by pathway competence, copper access, and metabolic state rather than by any single marker [28,289]. Serum copper and ceruloplasmin may still be useful as pharmacodynamic indicators of systemic copper perturbation, but they are unlikely to replace tumor-level biomarkers for predicting response [5,293]. None of these candidates has yet been prospectively validated as a predictive biomarker for cuproptosis-based treatment, so discussion in this area should remain hypothesis-generating rather than clinically definitive [[289], [290], [291]]. For a state-dependent therapy such as cuproptosis, patient stratification is therefore likely to depend on multiple parameters rather than any single marker. Transcriptomic cuproptosis scoring models derived from The Cancer Genome Atlas (TCGA) and related datasets have become increasingly common, but they remain largely correlative and often provide prognostic rather than predictive information [255,294,295]. Proteomic measurements may be more informative for key pathway components such as FDX1 and lipoylated proteins, since transcript abundance does not always reliably reflect functional protein levels [296]. Metabolomics offers a complementary advantage by more directly reflecting TCA-cycle activity, redox state, and glutathione balance [297]. Spatial transcriptomics and single-cell analysis also help reveal intratumoral heterogeneity that may be obscured in bulk measurements [253,298]. Clinically useful stratification will probably require an integrated measure of pathway competence, copper handling and metabolic state rather than a single gene. A major missing element, however, is a matched dataset linking such multi-omics profiles to actual response under cuproptosis-directed treatment. Without such data, machine-learning-based precision models remain speculative.

The longer-term goal extends beyond biomarker discovery to the more precise matching of patients with appropriate nanomedicine strategies. In practice, this requires assessment of tumor metabolic permissiveness, cuproptotic pathway integrity, copper influx and efflux status, and the suitability of the immune microenvironment for combination with checkpoint blockade or innate immune activation. From a translational perspective, these factors are valuable only if they help guide treatment decisions rather than remain part of a purely descriptive biomarker panel. Theranostic design may help make this approach more clinically actionable. For example, ^64^Cu-PET and complementary imaging platforms could assess tumor delivery and off-target distribution, provided that signal stability and tracer dissociation are validated [157,158,275]. In this way, patient selection can be linked more directly to treatment monitoring. A patient might be selected according to tumor biology, dosed according to image-informed delivery behavior, and monitored during treatment using the same or a closely related platform. Such an approach is likely to be more realistic than relying on baseline biomarkers alone [131].

Regulatory uncertainty is likely to be a significant challenge for many cuproptosis nanomedicines. Platform classification may vary with design, including classification as a drug, a combination product, or a hybrid system that incorporates diagnostic or nucleic acid therapy elements [263,299]. Because classification determines the review pathway together with the manufacturing, pharmacology, and safety evidence required for development, this variability has direct translational consequences. Although existing regulatory guidance for nanomedicine provides a useful reference, much of it was developed for lipid and polymer systems rather than inorganic copper-based platforms [299,300]. Cuproptosis nanomedicine may therefore face a familiar translational gap in which scientific innovation outpaces regulatory precedent [131]. Early and sustained regulatory engagement will be critical. Pre-investigational new drug meetings or equivalent scientific-advice procedures should clarify chemistry, manufacturing and controls requirements, biodistribution plans and toxicology strategies [131].

As of July 2026, to our knowledge, no cuproptosis-specific nanomedicine has entered clinical testing, but existing clinical experience with copper-modulating agents still provides useful context. DSF combined with copper supplementation demonstrates the clinical feasibility of pharmacological copper perturbation, although evidence of efficacy has so far remained limited [198,[301], [302], [303]]. Tetrathiomolybdate and related chelation strategies extend this perspective by showing that systemic copper depletion is likewise pharmacologically feasible and can be monitored through markers such as ceruloplasmin, even though its antitumor benefit has remained limited [293,304]. Together, these clinical experiences provide practical guidance for copper monitoring, dose adjustment, and safety oversight in future copper-elevating strategies. The phase III elesclomol trial cautions against developing copper-directed therapies in unselected populations, but it does not establish that biomarker selection alone would have produced clinical benefit [21,288]. This is an important warning for the cuproptosis field because even a mechanistically sound copper-directed therapy may fail clinically if it is developed without biomarker-guided stratification. First-in-human development should prioritize simple, scalable lead platforms supported by Good Laboratory Practice (GLP)-compliant toxicology and robust pharmacokinetic and biodistribution data. Early trials should incorporate analytically validated pharmacodynamic biomarkers that can demonstrate target engagement independently of tumor shrinkage. Given the mechanistic specificity and toxicological sensitivity of this field, early clinical development will need to emphasize proof of biology as much as proof of safety [31,131].

## 6 Remaining challenges and future directions

**Fig. 11:**
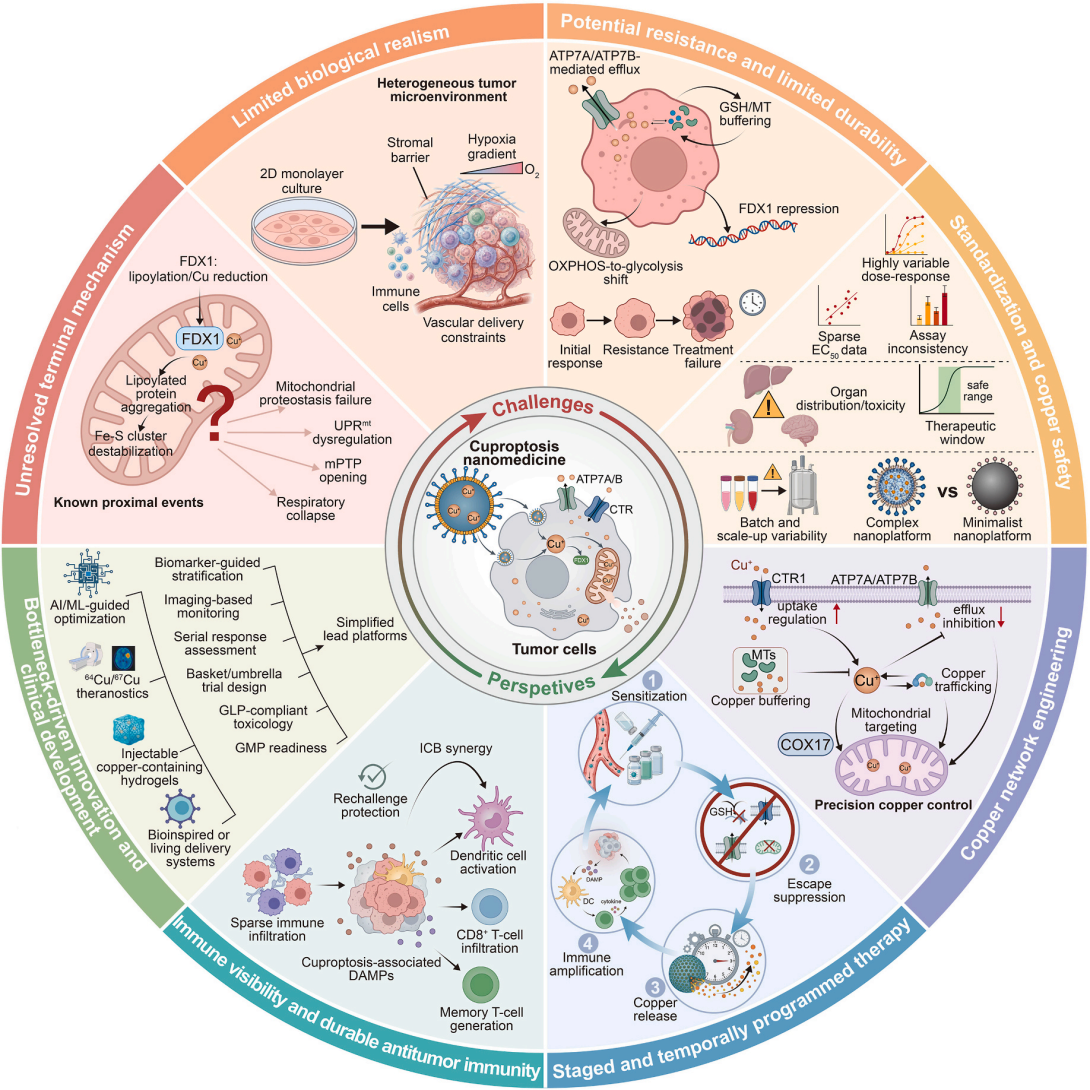
**Challenges and perspectives for cuproptosis nanomedicine in cancer.** Major challenges include the unresolved terminal mechanism of cuproptosis, limited biological realism of two-dimensional cultures, tumor microenvironment heterogeneity, adaptive resistance through ATP7A/ATP7B-mediated efflux, glutathione and metallothionein buffering, FDX1 repression and metabolic switching, together with variable dose responses, narrow therapeutic windows, organ toxicity, batch variability and excessive platform complexity. Future development should prioritize precision control of copper uptake, trafficking, buffering and mitochondrial delivery; staged therapy integrating sensitization, escape suppression, copper release and immune amplification; durable antitumor immunity; and clinically oriented platform development supported by biomarker-guided stratification, radiocopper theranostics, AI/ML-guided optimization, simplified lead formulations, GLP-compliant toxicology, GMP readiness and rational trial design. **Note:** AI, artificial intelligence; ATP7A/ATP7B, ATPase copper transporting alpha/beta; CD8^+^ T cell, cluster of differentiation 8-positive T cell; COX17, cytochrome *c* oxidase copper chaperone 17; CTR1, copper transporter 1; Cu, copper; Cu^+^, cuprous copper; DAMPs, damage-associated molecular patterns; DC, dendritic cell; EC_50_, half-maximal effective concentration; FDX1, ferredoxin 1; Fe–S, iron–sulfur; GLP, Good Laboratory Practice; GMP, Good Manufacturing Practice; GSH, glutathione; ICB, immune checkpoint blockade; ML, machine learning; mPTP, mitochondrial permeability transition pore; MTs, metallothioneins; OXPHOS, oxidative phosphorylation; UPRᵐᵗ, mitochondrial unfolded protein response; ^64^Cu, copper-64; ^67^Cu, copper-67.

Cuproptosis nanomedicine has developed rapidly, but its clinical translation remains at an early stage. Current studies encompass copper-containing nanomaterials, copper-ionophore formulations, metabolic sensitization and immunotherapy-oriented combinations. However, preclinical efficacy alone is insufficient to establish a clinically viable strategy. Translation will require selective and reproducible pathway engagement, quantitative pharmacology, biologically relevant models, and tractable platform design [31,248,[305], [306], [307], [308]]. The field should therefore shift from continued platform proliferation toward mechanism-specific validation, copper-network engineering, immune programming, and biomarker-guided clinical development (Fig. 11).

### 6.1 Mechanistic clarity and biological credibility

The terminal execution mechanism of cuproptosis remains incompletely defined. FDX1-associated copper activation, aggregation of lipoylated proteins and loss of Fe–S proteins are established proximal events [10]. However, the processes that convert these lesions into irreversible cell death remain uncertain. Candidate mechanisms include collapse of mitochondrial proteostasis, maladaptive mitochondrial unfolded protein response signaling, opening of the mitochondrial permeability transition pore and Fe–S-dependent respiratory failure [16,[307], [308], [309], [310], [311], [312]]. Resolving this terminal step will be important for identifying rational amplification points, resistance mechanisms and pharmacodynamic markers.

Future studies should prioritize dynamic in vivo reporters and longitudinal resistance models capable of resolving when and where cuproptosis occurs and how therapeutic escape develops. Genetically encoded reporters, organelle-resolved copper sensors, and circulating pharmacodynamic markers may provide more direct evidence of pathway engagement than endpoint staining alone [31,131,248,[305], [306], [307], [308]]. These tools should be applied in models that reproduce metabolic gradients, stromal barriers, immune interactions and heterogeneous nanoparticle delivery [16,249].

Resistance should also be studied as an evolving process rather than inferred from baseline biomarkers. Plausible escape mechanisms include ATP7A/ATP7B-mediated copper efflux, reinforced glutathione and metallothionein buffering, FDX1 repression and metabolic switching from oxidative phosphorylation toward glycolysis [17,47,55,141,145,242,254,255,310,311]. Longitudinal models should determine whether mechanism-matched interventions can prevent, delay or reverse these adaptations [308].

### 6.2 Quantitative rigor and shared standards

Progress requires a shared minimum dataset integrating biologically active copper exposure, pathway-specific validation and matched normal-tissue toxicity. Nominal nanoparticle dose is an inadequate pharmacological metric because it does not reflect intracellular copper availability, mitochondrial delivery or pathway engagement. Studies should therefore quantify intracellular and subcellular copper together with DLAT aggregation, Fe–S protein loss, copper-chelator rescue and genetic manipulation of FDX1 or the protein-lipoylation machinery.

Standardization is also needed for exclusion of alternative death pathways, combination analysis, biodistribution and organ-level safety. A core reporting framework should include copper speciation and release, serum stability, cellular and mitochondrial copper accumulation, cuproptosis competence, rescue controls, formal synergy analysis, pharmacokinetics, clearance and repeated-dose toxicity [31,131,248,249,305]. These shared standards would improve cross-study comparability and help distinguish genuine mechanistic advances from variation in formulation, exposure or assay design.

### 6.3 Simplicity, manufacturability, and copper-specific safety

Translational development will require greater emphasis on minimalist platform design. Multifunctional systems may combine copper delivery, targeting ligands, redox modulators, gene-silencing components, immune agonists, and biomimetic coatings, but every additional component increases analytical, manufacturing, and regulatory complexity [24,31,131,[152], [153], [154],^248^,^305^]. Each module should therefore address a defined biological barrier and provide a measurable contribution to therapeutic performance.

Lead platforms should favor mature carrier systems, reproducible composition, scalable synthesis and clinically meaningful advantages over simpler alternatives. Simplification should not be viewed as a loss of innovation, but as a prerequisite for robust characterization, batch-to-batch consistency and regulatory evaluation [313]. Early development should incorporate critical quality attributes, release specifications, stability testing and GMP-compliant manufacturing strategies rather than postponing these issues until late preclinical stages.

Copper-specific safety must be evaluated beyond acute tolerability. Because copper homeostasis is essential in normal tissues, repeated perturbation may cause hepatic, renal, neurological or systemic metabolic toxicity [[259], [260], [261]]. The liver requires particular attention because it is central to copper metabolism and nanoparticle sequestration, while the kidney and nervous system may become vulnerable during prolonged exposure or barrier disruption [129,263,267]. Repeated-dose studies should therefore assess tissue copper burden, recovery after treatment, organ function, mitochondrial injury, and delayed toxicity. The central safety question is whether therapeutic copper stress can remain spatially restricted and reversible outside the tumor.

### 6.4 Precision development and emerging opportunities

A promising direction is precision regulation of the copper network rather than indiscriminate copper overload. Coordinated control of copper uptake, trafficking, buffering, efflux, and mitochondrial routing may permit effective pathway engagement at lower total copper doses [17,31,305]. Moderate copper exposure combined with selective efflux suppression, controlled buffering attenuation, or improved mitochondrial delivery may offer a wider therapeutic window than dose escalation alone.

Temporal programming may further improve selectivity. Buffering depletion or efflux restriction may need to precede copper release, whereas immune amplification may be most effective after tumor-cell injury has generated antigens and danger signals. This supports staged strategies in which sensitization, escape suppression, copper activation and immune engagement are coordinated sequentially rather than incorporated as simultaneously active modules.

Cuproptosis-associated immune activation also warrants more rigorous development. Future studies should define the kinetics of DAMP release, mtDNA–cGAS–STING signaling, dendritic-cell activation, T-cell priming, macrophage remodeling and memory formation [306,308]. Functional evidence should include immune-cell depletion, checkpoint dependence, control of distant untreated tumors and resistance to tumor rechallenge. These experiments will determine whether cuproptosis can convert immunologically poorly responsive tumors into lesions that are more susceptible to immune checkpoint blockade or innate immune agonists.

Emerging technologies should be selected according to the translational barrier they resolve. Machine learning may support formulation optimization when sufficiently large and standardized datasets connect material properties with release, stability, biodistribution and toxicity [[314], [315], [316]]. Radiocopper theranostics using ^64^Cu and ^67^Cu may integrate whole-body imaging, dosimetry and radionuclide therapy while remaining directly relevant to copper biology [158,275,317,318]. Injectable copper-containing hydrogels may provide sustained locoregional exposure after surgery, whereas bioinspired or living delivery systems may improve penetration in poorly accessible tumors [164,[319], [320], [321]]. Their value should be judged by pharmacological performance and manufacturability rather than technological novelty alone.

Clinical development should be organized around biomarker-guided patient selection, imaging-based monitoring and serial pharmacodynamic assessment [287,[322], [323], [324]]. Early trials could use biomarker-enriched dose-escalation cohorts followed by pharmacodynamic expansion cohorts. Basket designs may become appropriate only after a tumor-agnostic biomarker has been prospectively validated [325]. Near-term priorities should therefore include robust pathway-verification criteria, resistant model systems, quantitative intracellular copper pharmacology and direct in vivo readouts. Longer-term progress will depend on a limited number of analytically tractable lead platforms supported by validated biomarkers, GLP-compliant toxicology packages and scalable manufacturing. Future advances are more likely to arise from evidence-based consolidation than from further increases in material complexity [131].

## 7 Conclusions

Cuproptosis is a distinct form of regulated cell death triggered by copper and linked to lipoylation-dependent mitochondrial dysfunction. Unlike nonspecific copper toxicity, cuproptosis depends on mitochondrial copper access, protein lipoylation and oxidative metabolism. This biological specificity gives cuproptosis therapeutic potential, but also means that successful development depends on rigorous mechanistic validation, appropriate patient selection, and precise control over delivery.

Nanomedicine offers a potential means of controlling the distribution, release and subcellular routing of copper. Small-molecule ionophores and free copper salts can perturb copper homeostasis, but they offer limited control over tumor accumulation, subcellular targeting, activation, and treatment monitoring. By contrast, well-designed nanoplatforms can integrate these functions within a single system and provide more precise control over copper delivery. Accordingly, the key opportunity is to link controllable tumor-site copper activation with pathway-competent tumor contexts in which mitochondrial stress can be converted into therapeutic benefit.

Further demonstrations of nonspecific cytotoxicity will add limited value unless they establish mechanism-specific pathway engagement and a therapeutic window. Future progress will depend instead on clearer mechanistic definition, stronger quantitative pharmacology, more relevant model systems, simpler and more manufacturable designs, and biomarker-guided strategies that can identify the tumors in which cuproptosis is not only inducible but also therapeutically meaningful. In this sense, future advances are less likely to come from increasingly complex platform design than from simpler, better-supported strategies that are more closely aligned with mechanistic understanding and translational needs.

Cuproptosis nanomedicine is therefore entering a stage in which mechanistic rigor, pharmacological definition, and translational feasibility matter more than conceptual novelty alone. The early phase of rapid growth established proof of concept and produced a wide range of material designs, but the next stage will depend on whether biologically meaningful problems can be solved in the right patients, with acceptable safety and a realistic path to manufacturing and clinical translation. If this shift can be achieved, cuproptosis nanomedicine may progress from a compelling mechanistic concept to a meaningful component of precision cancer therapy.

## Ethics approval and consent to participate

Not applicable. This research did not involve human participants, animals, or access to identifiable personal data.

## CRediT authorship contribution statement

**Dongsheng Fan:** Writing – original draft. **Jianxin Liu:** Project administration, Writing – original draft. **Fang Ren:** Investigation, Visualization. **Long Wang:** Investigation. **Jing Wei:** Validation. **Dalian Qin:** Investigation. **Xiaogang Zhou:** Conceptualization. **Jianming Wu:** Funding acquisition, Supervision. **Xiao Zou:** Conceptualization, Visualization. **Anguo Wu:** Conceptualization, Funding acquisition, Supervision, Writing – review & editing.

## Declaration of competing interest

The authors declare no conflicts of interest.

## Data Availability

No data were used for the research described in this article.
